# The CNS-Penetrant Soluble Guanylate Cyclase Stimulator CY6463 Reveals its Therapeutic Potential in Neurodegenerative Diseases

**DOI:** 10.3389/fphar.2021.656561

**Published:** 2021-05-24

**Authors:** Susana S. Correia, Rajesh R. Iyengar, Peter Germano, Kim Tang, Sylvie G. Bernier, Chad D. Schwartzkopf, Jenny Tobin, Thomas W.-H. Lee, Guang Liu, Sarah Jacobson, Andrew Carvalho, Glen R. Rennie, Joon Jung, Paul A. Renhowe, Elisabeth Lonie, Christopher J. Winrow, John R. Hadcock, Juli E. Jones, Mark G. Currie

**Affiliations:** ^1^ Cyclerion Therapeutics, Cambridge, MA, United States; ^2^ Ironwood Pharmaceuticals, Cambridge, MA, United States

**Keywords:** cGMP (cyclic GMP), nitric oxide, soluble guanylate cyclase (sGC), neurodegeneration, neuroprotection

## Abstract

Effective treatments for neurodegenerative diseases remain elusive and are critically needed since the burden of these diseases increases across an aging global population. Nitric oxide (NO) is a gasotransmitter that binds to soluble guanylate cyclase (sGC) to produce cyclic guanosine monophosphate (cGMP). Impairment of this pathway has been demonstrated in neurodegenerative diseases. Normalizing deficient NO-cGMP signaling could address multiple pathophysiological features of neurodegenerative diseases. sGC stimulators are small molecules that synergize with NO, activate sGC, and increase cGMP production. Many systemic sGC stimulators have been characterized and advanced into clinical development for a variety of non-central nervous system (CNS) pathologies. Here, we disclose the discovery of CY6463, the first brain-penetrant sGC stimulator in clinical development for the treatment of neurodegenerative diseases, and demonstrate its ability to improve neuronal activity, mediate neuroprotection, and increase cognitive performance in preclinical models. In several cellular assays, CY6463 was demonstrated to be a potent stimulator of sGC. In agreement with the known effects of sGC stimulation in the vasculature, CY6463 elicits decreases in blood pressure in both rats and mice. Relative to a non-CNS penetrant sGC stimulator, rodents treated with CY6463 had higher cGMP levels in cerebrospinal fluid (CSF), functional-magnetic-resonance-imaging-blood-oxygen-level-dependent (fMRI-BOLD) signals, and cortical electroencephalographic (EEG) gamma-band oscillatory power. Additionally, CY6463 improved cognitive performance in a model of cognitive disruption induced by the administration of a noncompetitive N-methyl-D-aspartate (NMDA) receptor antagonist. In models of neurodegeneration, CY6463 treatment increased long-term potentiation (LTP) in hippocampal slices from a Huntington’s disease mouse model and decreased the loss of dendritic spines in aged and Alzheimer’s disease mouse models. In a model of diet-induced obesity, CY6463 reduced markers of inflammation in the plasma. Furthermore, CY6463 elicited an additive increase in cortical gamma-band oscillatory power when co-administered with donepezil: the standard of care in Alzheimer’s disease. Together, these data support the clinical development of CY6463 as a novel treatment for neurodegenerative disorders.

## Introduction

Neurodegenerative diseases exact an immense toll on the lives of patients, their families, and healthcare systems across the world. Traditional treatment approaches have generally been focused on targeting pathogenic proteins and the damage they cause. In light of recent high-profile failures in Alzheimer’s disease (AD) clinical trials (Elmaleh et al., 2019), approaches addressing the broader aspects of neurodegenerative pathologies such as neuroinflammation, synaptic and neuronal dysfunction, and cerebrovascular dysregulation may provide a path forward for new therapies.

Nitric oxide (NO) is a gasotransmitter that binds to soluble guanylate cyclase (sGC) to produce the critical downstream messenger, cGMP, thereby regulating key homeostatic processes in the CNS and in peripheral tissues. Specifically, intracellular cGMP regulates vascular tone and regional blood flow, fibrosis, and inflammation and has been implicated in neuronal survival and function (Aissa et al., 2016; Garthwaite, 2019). Impairment of the NO-sGC-cGMP signaling pathway is associated with cardiovascular disease, diabetes, and pathogenesis of neurodegenerative diseases and has been observed in vascular dementia, AD, general cognitive impairment, and could lead to stroke (Bennett et al., 2009; Stephan et al., 2017; Casas et al., 2019a; Casas et al., 2019b). NO bioavailability and disrupted NO-sGC-cGMP signaling may be impaired by several mechanisms, including endothelial dysfunction and concomitant reduction in endothelial nitric oxide synthase (eNOS) activity, increased levels of the nitric oxide synthesis (NOS) inhibitor asymmetric dimethyl arginine, and increased oxidative stress and reactive oxygen species that react with NO (Lourenço et al., 2017). Endothelial cell loss and NO dysregulation are recognized as major contributing factors in neurodegenerative diseases, resulting in reduced blood flow, vascular leakage, and inflammation, along with synaptic dysfunction and neuronal loss (Toth et al., 2017).

Different mechanistic approaches can be considered to compensate for NO deficiency. Phosphodiesterase (PDE) inhibitors, sGC activators, and sGC stimulators are all small molecules that can increase cGMP levels. PDE inhibitors modulate cGMP signaling by inhibiting cGMP degradation and preserving the levels of cGMP produced. sGC activators and stimulators enhance cGMP signaling by increasing the production of cGMP at the source—a mechanism that may have a more robust effect on the cGMP pathway. sGC activators act on the oxidized, heme-free form of sGC, which is unresponsive to endogenous NO. In contrast, sGC stimulators are positive allosteric modulators that act on the native, reduced, heme-containing form of the enzyme to produce cGMP synergistically with endogenous NO. The activity of sGC stimulators may compensate for deficient NO bioavailability in neurodegenerative diseases and may in turn improve endothelial function, restore local blood flow, and improve neuronal function. CY6463 (formerly known as IW-6463) is an orally administered, clinical-stage, sGC stimulator designed to enter the CNS and is being investigated as a potential treatment for multiple neurodegenerative diseases (Rennie et al., 2019). Preclinical pharmacology studies of CY6463 utilized modalities that can also be used in clinical studies, including fMRI-BOLD imaging, quantitative electroencephalography (qEEG) analysis, cerebrospinal fluid (CSF) biomarker assessment, magnetic resonance spectroscopy (MRS), and cognitive assessments. Additional studies characterized the mechanism of action of CY6463. In rodent studies described here, CY6463 increased fMRI-BOLD signals, elevated qEEG gamma-band oscillatory power, and increased levels of cGMP in the CNS—in contrast to a CNS-restricted sGC stimulator that demonstrated a lack of target engagement and distinct pharmacology in the CNS. In models of CNS impairment in rats, CY6463 improved dendritic spine density, reversed brain metabolite N-acetyl-aspartate (NAA) + N-acetyl-aspartate-glutamate (NAAG) deficits, restored hippocampal long-term potentiation (LTP), increased neurotrophic factors such as phosphorylated cAMP-response element binding (pCREB) and brain-derived neurotrophic factor (BDNF), and improved behavioral task performance in pharmacologically impaired rats. Taken together, these data support the clinical development of CY6463 as a novel treatment for neurodegenerative disorders.

## Materials and Methods

### Animal Husbandry

Unless otherwise stated, rats and mice were housed individually in polycarbonate cages with filter tops, acclimated for at least 3 days prior to study start, under controlled conditions of temperature (22 ± 4°C) and a relative humidity of 30–70% and placed in a 12:12-h light-dark cycle room (lights on at 6:30 A.M.) at an AAALAC-accredited animal research facility. Unless otherwise noted, in mouse studies, C57BL mice (6–9 weeks old) were studied. Animals were allowed *ad libitum* access to water and standard rodent chow (Harlan Teklad, Indianapolis, IN; Irradiated Teklad Global 16%).

### Data Analysis

Unless noted, statistical significance was determined by analysis of variance (ANOVA). A significant main effect was followed with an appropriate post hoc test. An effect was considered significant if *p* < 0.05. Data are presented as the mean ± standard error of the mean (SEM) and were graphed and analyzed using GraphPad Prism (v 8).

### Measurement of cGMP Using the GloSensor Cell-Based Assay

Human embryonic kidney 293 (HEK293) cells expressing the cGMP sensor, GloSensor™ 40 F clone (Promega, Cat. # CS182801), were maintained in DMEM supplemented with fetal bovine serum (FBS) (10% final) and hygromycin B (200 μg/ml). For sGC activity assays, cells were seeded in DMEM with 10% FBS in a 50-µl volume at a density of 1.5 × 10^4^ cells/well in a poly-D-lysine coated, 384-well, white, flat-bottom plate (Corning, Cat. #35661). Cells were incubated overnight at 37°C in a humidified chamber with 5% CO_2_. The next day, medium was replaced with 2 mM GloSensor reagent (Promega, Cat. #E1291) at 40 µl/well in CO_2_-independent medium (Gibco Cat. #18045-088). Cells were treated for 90 (min) at 25°C to allow the reagent to equilibrate in the cells. Diethylenetriamine NONOate (DETA-NONOate) was added to the wells to make a 10-µM concentration for DETA-NONOate solution and test compound at final concentrations: 0.029, 0.114, 0.460, 1.83, 7.32, 29.29, 117.2, 468.8, 1875, 7500, and 30000 nM. After a 50-min incubation at room temperature, the luminescence signal generated by the interaction of intracellular cGMP with GloSensor 40 F protein was measured with Envision Multilabel Reader (Perkin Elmer, Model #2104-0010 A). Data were analyzed with a 4-parameter fit (log (agonist) vs. response − variable slope). The EC_50_, defined as the concentration at which a given compound elicits 50% of the maximal response was interpolated for CY6463 and vericiguat from the curve fit.

### Preparation of Rat Primary Neurons Culture

Neurons were isolated from Sprague Dawley rat embryos on embryonic Day 18. *Hippocampus* and cortex were dissected from the brains under a stereoscopic microscope. The tissues were chopped and washed gently once with 10 ml of Ca^2+^ and Mg^2+^ free HBSS (Corning, Cat. #21-022-CM) in a 15-ml conical tube. After washing, 5 ml of a solution consisting of 0.25% trypsin (Invitrogen, Cat. #15090-046) and 0.1% deoxyribonuclease I (DNase I, Sigma, Cat. #DN-25) were added to the tissues and incubated at 37°C for 15 min. Next, tissues were washed times with ice-cold HBSS. Next, 3 ml of a solution of 0.1% of DNase I was added. Tissues were then slowly pipetted 12 times using a glass Pasteur pipette, followed by centrifugation at 500 × g for 10 min. The resulting cell pellet was resuspended in culture medium (Neurobasal medium, Gibco, Cat. #21103-049), 2% of B27 supplement (Gibco, Cat. #17504-044), 0.5 mM L-glutamine (Corning, Cat. #25-005-Cl), 25 μM L-glutamic acid (Sigma, Cat. #G1251), and 1% penicillin/streptomycin (Gibco, Cat. #15070-063). The cell suspension was plated into poly-L-lysine coated 384-well plates (for cGMP assay) or 96-well plates (for pCREB assay) at 30,000 or 100,000 cells/well, respectively. 24 h after plating, half of the culture medium was removed and replaced with the culture medium described above without glutamic acid. Cells were maintained in a 37°C humidified incubator with 5% CO_2_ and used between Days through.

### cGMP Measurement in Rat Primary Neurons

Rat primary neurons were washed once with HBSS with calcium and magnesium and incubated with a solution containing 0.5 mM 3-isobutyl-1-methylxanthine (IBMX) in HBSS (80 uL/well) at 37°C for 15 min. A 5X stock (20 µl) of test compound with a fixed concentration of DETA-NONOate was added to the wells to make *x* nM concentration for test compound solution and 30 µM concentration for DETA-NONOate solution, where *x* is 1 of the following final concentrations 0.029, 0.114, 0.460, 1.83, 7.32, 29.29, 117.2, 468.8, 1875, 7500, and 30,000 nM. In previous experiments, incubation with DETA-NONOate elicits ∼600–1000-fold increase in cGMP or pCREB compared to wells without the NO donor; therefore, the following experiments describe the data from wells treated with DETA-NONOate. The cells were incubated for 20 min at 37°C. cGMP was measured in cell lysate by liquid chromatography with tandem mass spectrometry (LC-MS/MS) as follows. A standard curve was prepared in 10% acetic acid (aqueous) containing 13°C cGMP (Toronto Research Chemicals) as the internal standard. Cell lysates and standards were analyzed for cGMP and internal standard levels using a Thermo Vantage Triple Quadrapole LCMS in positive Ionization (ESI+) mode, linked to an Acquity UPLC (Waters Corporation, Milford, MA). The samples were loaded onto a Thermo Hypersil Gold 2.1 × 50 mm, 1.9-micron particle-size column. The mobile phase consisted of aqueous 0.1% formic acid (mobile phase A) and acetonitrile with 0.1% formic acid (mobile phase B). The flow rate was 0.750 ml/min. The gradient was held at 100% mobile phase A for 0.2 min, ramped to 50% mobile phase A by 0.3 min, and held for 0.7 min before returning to 100% mobile phase A by 0.8 min. The total run time was 1 min per sample. Data were fit using a 4-parameter fit (log (agonist) vs. response − variable slope). The EC_50_ for CY6463 and vericiguat were interpolated from the curve fit.

### pCREB Measurement in Rat Primary Neurons

Rat primary neurons were washed once with HBSS with calcium and magnesium and incubated with 90 μl HBSS for 30 min at 37°C. A 10X stock (10 µl) of test compound with a fixed concentration of DETA-NONOate was added to the wells to make *x* nM concentration for test compound solution and 10 µM concentration for DETA-NONOate solution, where *x* is 1 of the following final concentrations: 0.01, 0.1, 1, 10, 100, 1000, and 10000 nM. The cells were incubated for an additional 30 min at 37°C. The medium was removed, cells were lysed and levels of pCREB and CREB were determined according to Cisbio protocols (phospho-CREB [Ser133], Cat. #64CREPEG and total CREB, Cat. #63ADK052PEG). Plates were read using Envision instrument (PerkinElmer). pCREB/CREB ratios were determined and analyzed with a 4-parameter fit (log (agonist) vs. response – variable slope). The EC_50_ for CY6463 was interpolated from the curve fit.

### Compound Exposure and cGMP Concentration in CSF in Rats

Prior to the experimental day, 31 male Sprague Dawley (CD) rats with intracisternal cannulation (250–275 g at arrival from Charles River Laboratories, Wilmington, MA) were fasted overnight. On the experimental day, rats were randomly assigned to each experimental group and administered vehicle (0.5% methylcellulose, 0.2% Tween 80, and 1% hydroxypropyl methylcellulose in filtered water), CY6463 (0.1, 1.0, 3.0, or 10 mg/kg), or vericiguat (30 mg/kg). At 1 and 6 h after dosing, animals were anesthetized with isoflurane, and 20 µl of CSF was withdrawn and discarded to compensate for the volume of the cannula. Then, approximately 50 µl of CSF was collected into Eppendorf tubes containing 5 µl of glacial acetic acid and were frozen in liquid nitrogen. 1 h postdose, blood was collected into EDTA tubes; plasma was isolated by centrifugation and kept frozen until analysis. Rats recovered for 3 or 4 days between treatments. The procedure was repeated up to times. Timepoints were chosen based on the pharmacokinetic profile of the compounds. In a previous study to understand the pharmacokinetic parameters (data not shown) in rats, the Cmax was determined from the maximal observed concentration in plasma following oral administration of CY6463 and vericiguat at relevant doses.

Concentrations of CY6463, vericiguat, and cGMP were measured in plasma and CSF by LC-MS/MS in ESI + mode as follows. Calibrants (CY6463, vericiguat: 0.50–1000 ng/ml; cGMP:0.01–400 ng/ml) were prepared in rat plasma and artificial CSF (with 20% acetic acid and 0.05% bovine serum albumin) by serial dilution. After internal standard addition, samples were precipitated with acetonitrile, were vortexed, and then centrifuged before a nitrogen dry-down and were reconstituted with 0.1% formic acid in water. Samples were analyzed for CY6463, vericiguat, and cGMP levels using a SCIEX 6500 QTrap (SCIEX, Framingham, MA) coupled to a Waters Acquity UPLC (Waters Corporation, Milford, MA). The samples were loaded onto a Waters HSS T3 (2.1 × 50 mm, 1.8 µm) with an HSS T3 guard column. The mobile phase consisted of aqueous 0.1% formic acid (mobile phase A) and acetonitrile with 0.1% formic acid (mobile phase B). The flow rate was 0.4 ml/min. The gradient was held at 100% mobile phase A for 1.0 min, ramped to 20% mobile phase A by 2.5 min, ramped to 100% mobile phase B by 3.0 min, and held for 0.3 min before returning to 100% mobile phase A by 3.6 min. The total run time was 5.5 min per sample.

Data for individual samples were interpolated from standard curve values for each analyte. Statistical analyses of data collected at 1 and 6 h postdose were performed separately because samples could not be collected from all animals at both time points due to low CSF flow, port blockage, etc. For CY6463, statistical significance was determined by the Kruskal-Wallis test followed by the uncorrected Dunn’s test. For vericiguat, statistical significance was determined by an unpaired two-tailed *t* test.

### Quantification of cGMP Concentration in Mouse Brain

A total of 97 male mice (ages 7–9 weeks, Envigo RMS, Inc., Indianapolis, IN) were group housed at Cyclerion Therapeutics as described above.

Mice were administered either vehicle (0.5% methylcellulose, 0.5% Tween 80 in filtered water) or CY6463 (0.3, 1.0, 3.0, or 10 mg/kg) by oral gavage. At 30-min postdose, mice were deeply anesthetized with isoflurane and the brain was collected. The cerebrum was separated from the hindbrain, collected in a 15- ml tube, flash frozen by immersion into liquid nitrogen, and stored at −80°C until analysis.

CY6463 and cGMP levels were measured in brain homogenate by LC-MS/MS in ESI + mode as follows. Calibrants were prepared in mouse brain homogenate by serial dilution. After internal standard addition, samples were precipitated with acetonitrile and were vortexed. They were then centrifuged before nitrogen dry down and were reconstituted with 0.1% formic acid in water. Samples were analyzed for CY6463 and cGMP levels using a Sciex 5500 QTrap coupled to a Waters Acquity UPLC. The samples were loaded onto a Waters Phenomenex Kinetex XB C18, 3.0 × 100 mm, 5 µm (Part # 00D-4605-Y0). The mobile phase was performed as described above. The flow rate was 1.0 ml/min. The gradient was held at 100% mobile phase A for 0.5 min, ramped to 100% mobile phase B by 2.0 min, and held until 2.5 min before being returned to 100% mobile phase A by 2.6 min. The total run time was 3.5 min per sample. Protein concentration in the cerebrum sample was quantified using a BCA protein quantification kit.

### Effect of CY6463 and Vericiguat on Blood Pressure in Rats

Hemodynamics effects of CY6463 were evaluated in rats harnessed to a tether system and connected to physiological pressure transducers (Harvard Apparatus APT300) overnight for acclimation. A blood pressure (BP) acquisition system (PowerLab data acquisition, bridge amps, and LabChart [v 7] software, ADInstruments, Colorado Springs, CO; and Harvard Apparatus, Holliston, MA) was used to monitor and analyze the hemodynamic data. Specifically, mean arterial pressure (MAP) and heart rate (HR) were measured in conscious, tethered, male, Sprague Dawley rats with indwelling femoral artery catheters (FEMART CD rats, Charles River Laboratories, Wilmington, MA; 250–275 g, 9–10 weeks of age). On the day of study, animals were disconnected from the transducers and blood was collected. After reconnection, baseline arterial pressure was measured for 1 h. Rats were then dosed with vehicle (0.5% methylcellulose with 0.2% Tween 80 in deionized water) or a single concentration of CY6463 via oral gavage (vehicle *n* = 5; 0.3 mg/kg *n* = 3; 1.0 mg/kg *n* = 4; 3.0 mg/kg *n* = 6; 10 mg/kg *n* = 3; 30 mg/kg *n* = 2). Vericiguat was evaluated using the same protocol (vehicle *n* = 9; 3 mg/kg *n* = 3; 10 mg/kg *n* = 7; 30 mg/kg *n* = 8).

Hemodynamic effects of multiple doses of CY6463 were also evaluated in rats. Prior to dose initiation, animals were acclimatized overnight to the tether and pressure transducer system. On the first day of dosing, baseline hemodynamics were measured for 1 h. Animals were then administered either vehicle or 10 mg/kg CY6463. Measurement of hemodynamic parameters was conducted for 4 h postdose. Rats remained connected to the tethers overnight. On Day 2, after 1 h predose recording, animals received their second dose of treatment. After monitoring the animals through the 4 h postdose timepoint, they were disconnected from the tether and pressure transducer. Dosing continued once daily on Days 3 through 5. Postdose on Day 5, rats were again acclimatized to the tether and pressure transducer system overnight. On Day 6, 1 h predose recordings were collected and measurement of hemodynamic parameters was continued for 4 h postdose.

During the monitoring hours described above, BP and HR data were recorded at 1000 data points per second and then averaged into 1 h bins for analysis. Change from baseline MAP (Δ_B_MAP) was calculated using the recordings averaged over the 1 h predose period using Microsoft Excel for Microsoft 365. Vehicle-adjusted MAP (Δ_V_MAP) was calculated by subtracting Δ_B_MAP of the vehicle group from the Δ_B_MAP of each dose group. For single-dose Δ_B_MAP and HR parameters, significance, as compared to vehicle, was determined by two-way repeated-measures ANOVA followed by a Dunnett’s post hoc test when appropriate. In the analysis of hemodynamic parameters after multiple doses, significance was determined for treatment as compared to the vehicle by two-way mixed-effect analysis followed by an Uncorrected Fisher’s least significant difference (LSD) test.

To determine plasma CY6463 exposure, blood samples were collected predose on Days 2 and 6 and at 2 h postdose on Days 1, 2, and 6. All blood samples were collected in K_2_EDTA blood collection tubes, separated by centrifugation (13,000 x g, 5 min, 4°C), transferred to Eppendorf tubes, and stored at -80°C until analysis.

### Effect of CY6463 on BP in Mice

A BP acquisition system (Dataquest ART acquisition and analysis system [v 4.36], Data Sciences International [DSI], St. Paul, MN) was used to collect single-dose BP and HR data from conscious, freely moving mice using a surgically implanted radio-telemetry pressure transmitter (PA-C10, DSI). A total of 18 male mice (body weight 27–29 g, Charles River Laboratories, Wilmington, MA) were surgically implanted with radio-telemetry pressure transmitters (DSI, St. Paul, MN) and then housed under controlled conditions at Gateway Pharmacology Laboratories as described above and were fed PicoLab® Rodent Diet 20 (Ft. Worth, TX). After a surgical recovery period, they received vehicle (in 0.5% [weight/volume] methylcellulose, 1% hydroxypropyl methylcellulose, and 0.2% Tween 80 in deionized water) or a single concentration of CY6463 each study day (9:00–10:00 A.M.) via oral gavage. Mice were reused after a 2- to 3-days washout period between dosing.

BP and HR data were recorded every 3 min and averaged into 1 h bins for analysis. Change from baseline MAP (Δ_B_MAP) was calculated using the predose baseline averaged over the 2 h period prior to dosing. Significance as compared to the vehicle for change from baseline MAP and HR data was determined by a two-way repeated-measures ANOVA followed by a Dunnett’s multiple comparisons test when appropriate. Vehicle-adjusted MAP (Δ_V_MAP) was calculated by subtracting Δ_B_MAP of the vehicle group from the Δ_B_MAP of each dose group. The 6 h average Δ_B_MAP was tested for significance by one-way ANOVA followed by a Dunnett’s multiple comparisons test.

### Quantification of fMRI-BOLD Response After CY6463 and Vericiguat Dosing

A total of 48 male CD rats (8–9 weeks, 275–300 g, Charles River Laboratories Wilmington, MA) were housed at Ekam Imaging Laboratories (Boston, MA) under the conditions described above. To reduce the stress associated with a head restraint, they were acclimated (4–5 consecutive days) to the restraining system (head holder and body tube; Animal Imaging Research, Holden MA) for 1 week prior to imaging. The restraining system included padded head support to obviate the need for ear bars, which reduces animal discomfort and minimizes motion artifacts. Rats were briefly anesthetized with 2–3% isoflurane while being secured into the head holder. When rats were again fully conscious, the imaging system was placed into a black opaque “mock scanner” box for 30 min, along with a tape-recording of the MRI pulse sequence to simulate the bore of the magnet and the imaging protocol.

Animals were then scanned at 300 MHz using a quadrature transmit/receive volume coil built into the rat head holder and restraining system for awake animal imaging. fMRI-BOLD experiments were conducted using a Bruker Biospec 7.0 T/20-cm UltraShield Refrigerated horizontal magnet (Bruker, Billerica, MA) and a 20-G/cm magnetic field gradient insert capable of a 120 μs rise time. At the beginning of each imaging session, a high-resolution anatomical data set was collected using a rapid acquisition with relaxation enhancement (RARE) pulse sequence (22 slice; 1.0 mm; field of view [FOV] 3.0 cm; matrix size 256 × 256; repetition time [TR] 2.5 s (s); echo time [TE] 12 milliseconds (ms); number of excitations (NEX) 2; 3 min acquisition time). Functional images were acquired using a multi-slice half-Fourier acquisition single-shot turbo spin-echo (HASTE) pulse sequence. A single scanning session acquired 22 slices, 1.0-mm thick, every 6.0 s (TR), using an effective TE of 48 ms, FOV 3.0 cm, matrix size 96 × 96, NEX 1, and repeated 350 times, for a total scanning time of 35 min. The in-plane pixel resolution was 312 μm^2^.

On the day of imaging, rats were lightly anesthetized with 2–3% isoflurane, fixed with a tail vein catheter, secured into the awake rodent imaging device, and positioned in the magnet. Rats recovered from anesthesia before the start of imaging. To deliver the drug remotely during the imaging session, a poly-ethylene tube (PE-20) approximately 30 cm in length was connected to the intravenous catheter. At the start of image acquisition, vehicle (10% PEG400, 25% of [20% Solutol HS 15 in Water], 65% DPBS), CY6463 (1.0 mg/kg), or vericiguat (3.0 mg/kg) was infused over 1 min into the tail vein. After administration, maximal drug concentrations were assumed to be reached immediately. The selected doses of CY6463 and vericiguat were expected to elicit a similar lowering of BP.

Data were coregistered to a mean functional image using SPM8’s coregistrational code with the following parameters: Quality: 0.97, Smoothing: 0.35 mm, and Separation: 0.5 mm. Gaussian smoothing was performed with an FWHM of 0.8 mm. Images were aligned and registered to the 3D rat brain atlas, segmented, and labeled with 171 discrete anatomical regions.

Using voxel-based analysis, the percent change in BOLD signal for each independent voxel was averaged for all animals. Each scanning session consisted of 350 data acquisitions (whole-brain scans) having a period of 6 s each, for a total lapse time of 35 min. The control window was the first 50 scan repetitions (5 min baseline), while the treatment stimulation window was scans 50 to 350 (min 5 through 35). Statistical t tests were performed on each voxel (∼15,000 in number) of each animal within their original coordinate system, with a baseline threshold of 2% BOLD change to account for normal fluctuation of BOLD signal in the awake rodent brain (Brevard et al., 2003). As a result of the multiple *t* test analyses performed, a false-positive detection controlling mechanism was introduced (Genovese et al., 2002). The results compared min 20 through 30 of imaging (acquisitions 200–300) to the 5 min baseline (acquisitions 1–50). Volume of activation was compared across experimental groups using the non-parametric Kruskal–Wallis test. Post hoc analyses were performed using a Wilcoxon rank-sum test.

### Quantification of qEEG Response by CY6463 and Vericiguat

A total of 12 adult male Sprague-Dawley rats (270–300 g, Envigo, Indianapolis, IN) were housed at PsychoGenics, Inc. (Paramus, NJ) as described above. Rats were implanted with DSI Telemetry devices (F40-EET) in standard 2-channel (lead pairs) configuration: Frontal/Parietal and neck electromyography (EMG), and single housed after surgery. After 7–10 days of surgical recovery, animals were placed in cages positioned over a DSI receiver for recordings and habituated to dosing (oral vehicle dosing; 0.5% methylcellulose and 0.5% Tween 80) for 5 days before the study start. Animals were tested twice weekly in a single-dose crossover design, with a 2- to 3-days washout period between doses. On each testing day, animals received either vehicle, CY6463 (0.1, 1, or 10 mg/kg), or vericiguat (0.3, 3, or 30 mg/kg) via oral gavage 10 min before 8:00 A.M. and 10 min before 8:00 P.M. Data were recorded continuously from 2 h prior to the first ante meridiem (A.M.) dose until 24 h after the second post meridiem (P.M.) dose.

Spectral analyses were performed by Cerridwen Software (NexStep Biomarkers, Madison, WI). During this process, adjacent non-overlapping recording segments of 30 s were subjected to an FFT to generate measures of spectral power (uV^2) for each of 1 Hz sub-bands (0–125 Hz range). These segments were summed in the time domain (summing 60 time segments to yield 600 s) to yield a cumulative power in each 600 s epoch. Additionally, any 30-s segment that contained an artifact was excluded from these calculations. A "relative power" for each 1-Hz sub-band (in a 30-s epoch) was then calculated by quantifying the ratio of the power for each 1 Hz sub-band by the "total power" (i.e., power [n Hz bin]/total power), defined as the sum of power across all frequency bands within a 600-s epoch. "Spectral band" power was then calculated as the sum of relative power for each 600‐s epoch between start/stop frequencies (e.g., 1–4 Hz). During the aggregation process, data were represented as time relative to the dose administration (0 s). For each recording, all data points were represented as a fold change from a "baseline" average of each "measure" (ie, each spectral band, each band ratio power, and each relative power). "Baseline" is defined as the average power in a 1-Hz sub-band, a band (e.g., delta), or a band ratio calculated for the 2-h recording period prior to treatment. After each recording was normalized to the dosing time and/or pre-drug baseline average, data were averaged across animals within each study arm. Spectral analyses included quantifying the relative spectral power per recording per rat for the traditional EEG bands (delta 1–4 Hz, theta 4–8 Hz, alpha 8–12 Hz, beta 12–30 Hz, and gamma 30–50 Hz). Data for each spectral band and each band ratio were averaged for 2-h bins beginning immediately after vehicle or compound administration and were used to examine the dose-dependent effects on qEEG for each compound. Data from rats treated with CY6463 or with vericiguat were compared to vehicle-treated rats using a two-way ANOVA followed by Dunnett’s post hoc analysis. Guided by when each compound should have achieved C_max_, the effects of CY6463 were examined 0–4 h after either the A.M. or P.M. dosing, and the effects of vericiguat were examined 4–8 h after A.M. or P.M. dosing. The effects of each compound and dose level on qEEG measures were assessed when animals exhibited waking. An awake state was defined using Cerridwen software’s decision tree algorithm, adapted from Johns et al., 1977.

### Quantification of qEEG Response by CY6463 and Donepezil

In total, 12 male Sprague-Dawley rats (∼300–350 g, Envigo, Indianapolis, IN) were studied under the same housing conditions and implanted with DSI telemetry devices (F50-EEE) in a 3-channel [lead pairs] configuration: frontal cortical, fronto-parietal, and neck EMG, as described above. Animals were tested twice weekly in a single-dose crossover design with a 2- to 3-day washout period between doses. On each testing day, within 10 min before 8:00 A.M. or 8:00 P.M., animals received CY6463 (10 mg/kg, oral) alone or in combination with donepezil (1 mg/kg, subcutaneous), donepezil (1 mg/kg, subcutaneous), or vehicle (oral 0.5% methylcellulose and 0.5% Tween 80). Telemetry data were recorded for 4 h predose and for 6 h postdose. Data for each spectral band were averaged. qEEG data acquisition and analysis was done as described above. Data were analyzed by two-way ANOVA followed by Tukey’s HSD post hoc analysis.

### Effect of CY6463 on a Novel Object Recognition Task

Eighty-three male Long-Evans rats (8–9 weeks; 275–299 g at arrival from Envigo, Indianapolis, IN) were housed 2 per cage at PsychoGenics as described above. Animals were randomly assigned into treatment groups (*n* = 15–16/group). All experiments were conducted during the light cycle.

The novel object recognition (NOR) test was conducted in an open-field arena (40 × 40 cm) placed in a sound-attenuated room under dimmed lighting. Each rat was tested separately, and care was taken to remove olfactory/taste cues by cleaning the arena and test objects with 70% alcohol between trials. All training and testing trials were video-recorded and scored by an observer blinded to treatments. On Days 1 and 2 (habituation), rats freely explored the arena with no objects inside for a 5-min period. On Day 3 (training and testing), rats were administered either vehicle (0.5% methylcellulose, 0.2% Tween 80 and 1% HPMC in filtered water) or CY6463 (0.01, 0.1, or 1.0 mg/kg) by oral gavage, 60 min prior to training. One group of rats was administered galantamine (1 mg/kg, intraperitoneal injection [IP]) 15 min prior to training. MK-801 (0.1 mg/kg, IP) or vehicle (saline, IP) was administered 15 min prior to training. For training, identical objects were placed in the test arena; the rat was then placed into a specific location of the test arena, always in the same orientation. The rat could freely explore both identical objects for 3 min and then was returned to their home cage. A total of 1 h after training, the testing trial began in which each rat was placed back into the test arena for 5 min in the presence of familiar object and novel object. Time spent exploring each object was recorded during the 5-min interval. The presentation order and position of the objects (left/right) in the testing trial were both randomized between rats to prevent bias from order or place preference.

Data are expressed as Recognition Index (RI), which is defined as the ratio of the time spent exploring the novel object divided by the total time spent exploring both objects (Novel/(Familiar + Novel) × 100%). Data were analyzed using 1-way ANOVA followed by Dunnett’s post hoc test on 0- to 1-, 0-3-, and 0-5 min intervals, separately. Animals with an overall object exploration time <10 s in the 5-min test session were eliminated; rats with an RI >90% or <30% were also eliminated because it suggested strong (non-memory) bias between objects. Any statistical outlier that fell above or below SDs from the mean were excluded from the final analysis. With these criteria, <3 rats were eliminated from each experimental group; the final number for analysis is reflected above.

### Effect of CY6463 on LTP in a Huntington’s (R6/2) Mouse Model

Adult (11- to 12-week old) R6/2 mice and their wild-type (WT) littermates (Beaumont et al., 2014) (Jackson Laboratory, Bar Harbor, ME) were group housed at Neuroservice (Aix-en-Provence, France) and studied in accordance with French and European legislations for animal care. Tissue was collected as previously described (Graef et al., 2015). Brains were removed and soaked in ice-cold oxygenated buffer (95% O_2_–5% CO_2_; KCl 2, NaH2PO4 1.2, MgCl_2_ 7, CaCl_2_ 0.5, NaHCO_3_ 26, Glucose 11, and Saccharose 250 [in mM]). Hippocampal slices (350 μm thickness) were prepared using a McILWAIN tissue chopper and incubated at room temperature for at least 1 h in ACSF (NaCl 126, KCl 3.5, NaH2PO4 1.2, MgCl2 1.3, CaCl2 2, NaHCO3 25, and Glucose 11 [in mM]). Slices were continuously perfused with ACSF (bubbled with 95% O_2_–5% CO_2_) during experiments at the rate of 3 ml/min, with a peristaltic pump (microelectrode array [MEA] chamber volume: ∼1 ml). Complete solution exchange in the MEA chamber was achieved 20 s after the switch of solutions. The perfusion liquid was continuously preheated at 37°C using a heated-perfusion cannula (PH01, MultiChannel Systems [MCS], Reutlingen, Germany) just before reaching the MEA chamber. The temperature of the MEA chamber was maintained at 37 ± 0.1°C using a heating element located in the MEA amplifier head-stage. All data were recorded with an MEA set-up (Steidl et al., 2006) commercially available from MCS that consisted of a 4-channel stimulus generator and a 60-channel amplifier head-stage connected to a 60-channel analog/digital card. Software for stimulation, recording, and analysis was commercially available from MSC (MC Stim II and MC Rack). All experiments were carried out with 3D MEA (Qwane Biosciences, S.A., Lausanne, Switzerland) consisting of 60 tip-shaped electrodes spaced 100 μm apart. Prior to recording, a 350-μm hippocampal slice was placed on the MEA (electrodes spaced by 100 μm). One electrode was selected to stimulate Schaeffer collaterals at the CA3/CA1 border such that stimulation by this electrode triggered fEPSP in the Stratum Radiatum (SR). The stimulus consisted of a monopolar biphasic current pulse (−250 μA for 60 μs followed by +250 μA for 60 µs) applied at 30 s intervals. fEPSPs were recorded for 10 min in control conditions (to verify the baseline steadiness) followed by a 15 min exposure to 7, 46, or 308 nM CY6463 (or 25 min in the presence of vehicle only, for control slices). After a 10 min control period in the continuous presence of CY6463, LTP was induced by a single train of 10 bursts, each consisting of 4 stimuli at 100 Hz, applied at a 200 ms interval (Theta-Burst Stimulation). Potentiation of synaptic transmission was then monitored over an additional 60 min period in the continuous presence of the compound. Because fEPSP results from glutamatergic synaptic transmission consecutive to afferent pathway stimulation, 10 μM NBQX (2,3-dioxo-6-nitro-7-sulfamoyl-benzo [f]quinoxaline, an antagonist of the AMPA receptor) was perfused on the slice at the end of each experiment, which validated the glutamatergic nature of synaptic transmission and subtracted background noise at an individual electrode level (data not shown). Each compound was evaluated on at least 10 slices from at least different R6/2 mice, recorded in parallel with 10 vehicle-treated R6/2 mice slices and vehicle-treated WT mice control slices. For basal transmission and LTP experiments, the fEPSP amplitude of each electrode was normalized as a function of the mean-averaged amplitude recorded over the 10-min control period. Normalized fEPSP values were first averaged for each slice, and then for all the slices in the same experimental conditions. The normalized fEPSP mean values (±SEM) were plotted as a function of time. Data from the last 20 min of the recording period were analyzed using 2-way repeated-measures ANOVA without Geisser-Greenhouse correction, followed by an uncorrected Fisher’s LSD.

### Measurement of Inflammatory Markers in the Plasma and BDNF in the Brains of Diet-Induced Obese Mice

Male diet-induced obese (DIO) and lean mice (15 weeks old; Taconic, Rensselaer, NY) were singly housed under thermoneutral conditions (29 ± 1°C) at Cyclerion Therapeutics. DIO mice were randomized into groups of similar body weight to receive either a high-fat diet (HFD; control) (Research Diets D12492i, *n* = 9) or HFD formulated with CY6463 to achieve a daily dose of 0.5 mg/kg (38.8 mg CY6463/kg of HFD, *n* = 9), 3 mg/kg (128.6 mg CY6463/kg of HFD, *n* = 9), or 10 mg/kg (386.2 mg CY6463/kg of HFD, *n* = 9) for 6 weeks. Lean mice received control chow for 6 weeks (Harlan 7912/Teklad LM-485; *n* = 9).

Body weight and food intake were measured every 3–4 days. 6 weeks after treatment initiation, non-fasted terminal blood and organs were collected. Each animal was sedated with isoflurane and exsanguination was performed via cardiac puncture. Whole blood was collected into EDTA tubes containing 20 μl Roche complete (protease inhibitor cocktail, Cat. #11 697 498 001), prepared at 50x. Plasma was aliquoted into 1.5-ml microfuge tubes for analysis of cytokines and was diluted 5-fold prior to analysis for the cytokine levels using BioRad Bio-Plex Pro 34-plex Mouse Chemokine Panel (Cat. #12002231; Standard Lot # 64084602; Control Lot #64084668) and processed according to manufacturer’s instructions. Data were acquired using Luminex MAGPIX (Bio-Rad, Hercules, CA). Bio-Plex Manager software was used to process data.

Brains were harvested and hypothalamus, hippocampus, and dorsal-vagal complex (DVC) of the hindbrain were microdissected, placed in individual 1.5-ml microfuge tubes, frozen on dry ice, and stored at −80°C until analysis. Mouse hindbrain BDNF protein concentrations were determined using the Quantikine Free BDNF ELISA kit (R&D Systems: Cat. #DBD00) following manufacture guidelines. To analyze gene expression levels, brain tissues (hippocampus and hypothalamus) were homogenized and processed using a QuantiGene sample processing kit (Affymetrix Cat. #QS013) in accordance with the manufacturer’s instructions. Gene expression in the tissues was measured using a QuantiGene 2.0 Plex Assay kit (Affymetrix Cat. #QP1013) and a multiplex gene panel (Thermo/Fisher, Plex Set #QGP-150-M18033001) following manufacturer guidelines. Analytes were measured using Luminex MAGPIX (Bio-Rad, Hercules, CA). Median fluorescence intensity was generated for each gene target and normalized to the geometric mean expression of housekeeping genes (Ppib, Rpl19, and Polr2a), which were chosen to match the target transcript abundance. Body weight and food intake were analyzed with a two-way repeated-measures ANOVA. Plasma proteins and gene expression were analyzed with a one-way ANOVA; significant differences were followed up with a Fisher’s LSD, comparing the CY6463-treated groups to the HFD control group.

### Effect of CY6463 on Neurometabolites by Magnetic Resonance Spectroscopy

A total of 64 Wistar rats (59 aged 23 months; 15 aged 2–3 months) were housed at Charles River Laboratories under the conditions described above. Aged rats were randomized to achieve treatment groups of similar body weight. For 14 days, they either received control diet (Research Diets D11112201, *n* = 18) or diet formulated with CY6463 to achieve a daily dose of either 0.3 mg/kg (16.4 mg CY6463/kg prepared in control diet, *n* = 19) or 1 mg/kg (54 mg CY6463/kg prepared in control diet, *n* = 20). Young rats received control diet (*n* = 15). Prior to imaging, rat in the vehicle group and rat in the 0.3 mg/kg CY6463 group died; the final number for analysis is reflected above.

Fourteen days after initiation of compound administration, MRS analysis and MRI acquisition (for anatomical reference) was performed in a horizontal 11.7-T magnet with bore size 160 mm that was equipped with a gradient set capable of maximum gradient strength of 750 mT/m and was interfaced to a Bruker Avance III console (Bruker Biospin GmbH, Ettlingen, Germany). A volume coil (Bruker Biospin GmbH, Ettlingen, Germany) was used for transmission and a surface phased array coil was used for receiving (Rapid Biomedical GmbH, Rimpar, Germany). Isoflurane-anesthetized rats were fixed to a head holder and positioned in the magnet bore in a standard orientation relative to gradient coils. For anatomical reference, MRIs of hippocampus Turbo-RARE images were collected using a field of view 25 × 25 mm^2^ and matrix of 256 × 256.

^1^H-MRS data were collected using the same experimental setup. A voxel was placed bilaterally in the hippocampus of the rat based on the anatomical images collected as described above. Automatic 3D gradient-echo shimming was used to adjust B0 homogeneity in the voxel. The water signal was suppressed using variable power radiofrequency pulses with optimized relaxation delays to obtain B1 and T1 insensitivity. A point-resolved spectroscopy sequence (TE = 11 ms) combined with outer volume suppression was used for the pre-localization. Three blocks were used and were interleaved with water suppression pulses. Data were collected by averaging 512 excitations (frequency corrected for each average) with TR of 2 s, number of points 4096, and spectral width 5 kHz. In addition, a reference spectrum without water suppression was collected from the identical voxel using the same acquisition parameters. Peak areas for metabolites were analyzed using LCModel (Stephen Provencher Inc., Oakville, Canada) and results were given relative to water content in tissue. Missing values for any metabolite concentrations were due to exclusion criteria on Cramer-Rao lower bounds >20% accuracy on estimation. Differences among means were analyzed by using a 1-way ANOVA followed by Dunnett’s test.

### Quantification of Spine Density in Mouse Models of Neurodegeneration

Male aged (63 weeks old) and young (6–7 weeks old) mice (Jackson Laboratory) were habituated to the vivarium for 1 week (aged mice) and 7 weeks (young mice) before study initiation. Mice were group housed at Cyclerion Therapeutics, as described above. Aged mice were randomly assigned to 1 of 2 experimental groups to receive control chow (Research Diets, New Brunswick, NJ, Cat. #D11112201; *n* = 7) or control chow formulated with CY6463 (71.6 mg CY6463/kg of chow; *n* = 7) for 18 weeks. Young mice received control chow for 18 weeks (*n* = 6). Blood samples were collected throughout the experiment to quantify CY6463 concentration. After 18 weeks of treatment, brains were paraformaldehyde fixed and processed to quantify hippocampal spine density.

For 3 months at PsychoGenics, 16 APP_HET PS1_HET female mice (24 weeks old) were treated with control chow (*n* = 8) or CY6463-formulated chow (214.4 mg CY6463/kg of chow; *n* = 8). Littermate control mice (APP_WT PS1_WT; *n* = 8) were treated with control chow. Mice were group housed as described above. Blood samples were collected throughout the experiment to quantify CY6463 concentration. After 12 weeks of treatment, brains were paraformaldehyde fixed and processed to quantify hippocampal spine density.

For both studies, tissue was processed by Afraxis, Inc. (San Diego, CA) as follows. Brains were sectioned coronally using a tissue vibratome (300 μm; Leica VT1000). DiI micro-tungsten particles were delivered by gene gun (110 psi) through a polycarbonate filter (3 μm; Millipore). Labeled sections were maintained for 48 h in 0.1 M PBS at room temperature to permit dye diffusion across labeled neurons. Finally, sections were slide mounted (Prolong Gold, Thermo Fisher Scientific) and imaged using super-resolution laser-scanning confocal microscopy (Zeiss LSM800/880, Airyscan). Hippocampal CA1 neurons were imaged using a 63X objective (1.42 NA) to scan individually labeled neurons at high resolution (scan resolution = 0.06 μm/pixel; axial resolution = 0.06 μm/focal step). Dendritic spine morphology was analyzed from samples taken from the first 50 µm of the secondary apical dendrites (measured from the branchpoint) from dorsal hippocampus CA1 neurons. A minimum of samples per mouse was measured for each segment. Blind deconvolution (AutoQuant Software, Media Cybernetics, Rockville, MD) was applied to raw 3D digital images, which were then analyzed for spine density and morphology by trained analysts. Individual spines were measured manually for head diameter, length, and neck thickness from image Z-stacks using custom-built Afraxis ESP (enhanced spine profiling) software. Each dendrite was analyzed by (on average) independent analysts who were blinded to all experimental conditions (including treatment, brain region, and cell type). For spine density and spine morphological classification, data across analysts were averaged to report data for each dendrite. Data were analyzed for each spine morphological classification using a one-way ANOVA followed by a Dunnett’s post hoc test to compare the CY6463-treated mice to the young vehicle-treated mice.

## Results

### *In vitro* Characteristics

**FIGURE 1:**
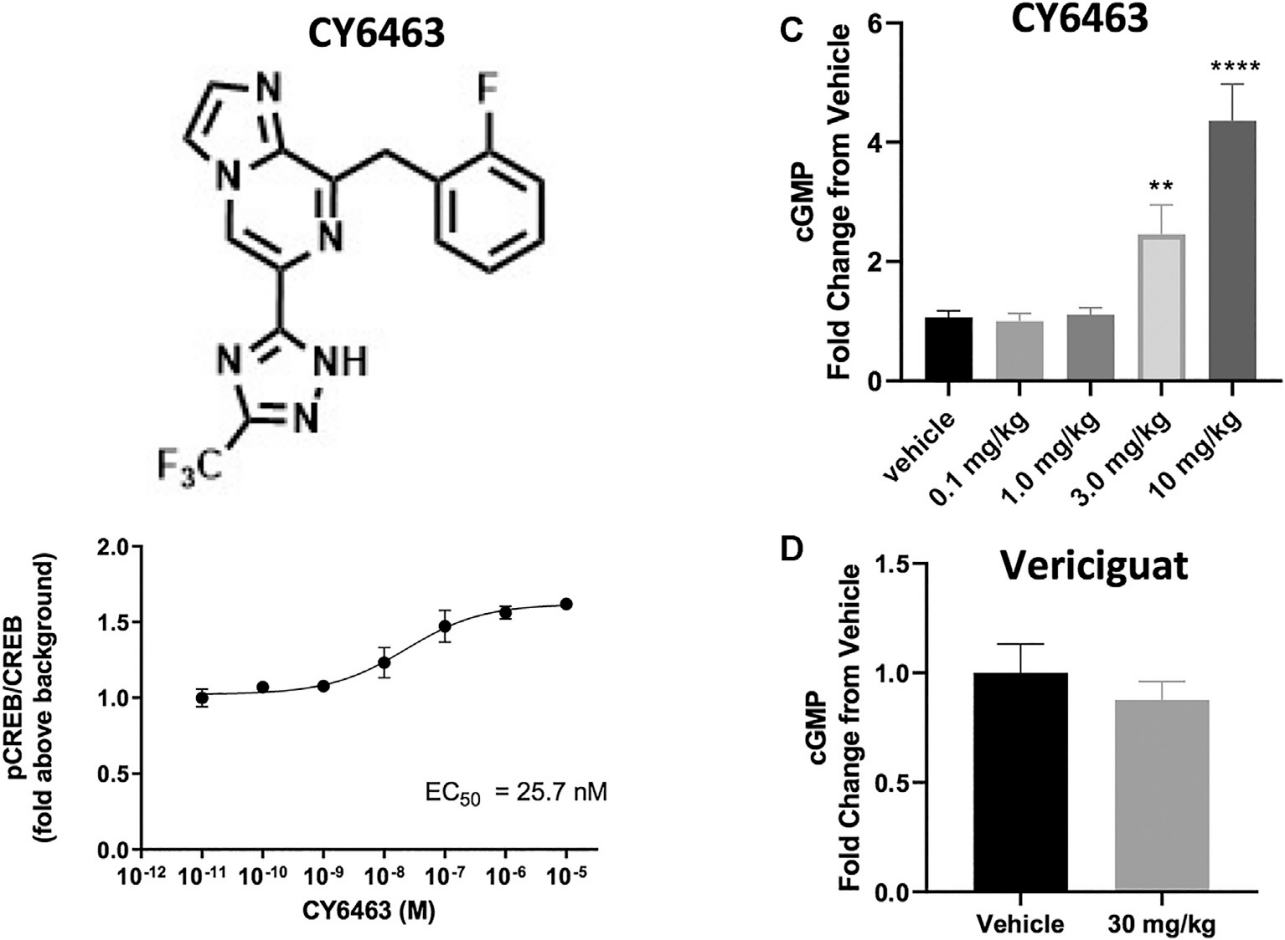
**(A)**, Chemical structure of CY6463 (8-(2-fluorobenzyl)-6-(3-(trifluoromethyl)-1*H*-1,2,4-triazol-5-yl)imidazo [1,2-*a*]pyrazine). **(B)**, Effect of CY6463 on CREB phosphorylation in rat primary neurons. Representative concentration response of CY6463 as measured by cellular phosphorylation of CREB in the presence of 10 µM DETA-NONOate in rat primary neurons. Data are normalized to vehicle control. Each data point represents the mean from three replicates. **(C)**, concentrations of cGMP in the CSF were higher in rats dosed orally with 3 or 10 mg/kg CY6463. (*p* = 0.0057 for the 3-mg/kg dose and *p* < 0.0001 for the 10-mg/kg dose; Kruskal-Wallis test followed by the uncorrected Dunn’s test). **(D)**, cGMP levels in the CSF in rats dosed orally with 30 mg/kg vericiguat were similar to those dosed with vehicle. **p < 0.01, ****p < 0.0001. All data are expressed as mean ± SEM.

In the presence of DETA-NONOate, CY6463 (Figure 1A) was more potent than vericiguat at stimulating sGC in the cell-based cGMP GloSensor® luciferase assay and in rat primary neurons (Table 1). CY6463 stimulated the phosphorylation of CREB in a concentration-dependent fashion in rat primary neurons, as shown by the representative curve (Figure 1B), with an average EC_50_ of 25.7 nM (95% CI, 0.6–149.3 nM).

**TABLE 1:** EC_50_ values for CY6463 and vericiguat.

	CY6463		Vericiguat	
	EC_50_ (nM)	N	EC_50_ (nM)	N
GloSensor™ 40 F cGMP	66 ± 6	33	263 ± 74	7
Rat primary neurons	46 ± 9	5	231 ± 53	5

GloSensor luciferase assay and cGMP assay of rat primary neurons in the presence of 10 or 30 µM DETA-NONOate, respectively. Values are means ± SEM of data from 5 to 33 independent experiments.

### CY6463 Elicited Increases in cGMP in the CNS

CY6463 and vericiguat were both found to be orally bioavailable and reached maximum concentration (C_max_) at 1 and 6 h in the rat, respectively. Plasma concentrations 1 h after oral administration were corrected using plasma protein binding (Waters et al., 2008) to determine the unbound distribution coefficient (K_p,uu_) as a ratio of CSF concentration over unbound plasma concentration (de Lange, 2013). For CY6463, the K_p,uu_ was 0.86, indicating passive permeability across the blood-brain barrier in the rat without active efflux by transporters. For vericiguat, the K_p,uu_ was indicating minimal partitioning into the CSF, likely due to efflux as vericiguat is a substrate for P-glycoprotein and breast cancer-resistance protein efflux transporters (Boettcher et al., 2020). While these calculations do not represent the equilibrium at the steady state, CY6463 can be considered as being a fully CNS penetrant sGC stimulator, and vericiguat to have limited exposure in the CNS. As expected with sufficient CNS exposure, 1 h after dosing, CY6463 increased CSF cGMP in rats (H (5) = 59.73, *p* < 0.0001) where rats administered 3 and 10 mg/kg CY6463 had significantly higher levels of cGMP in the CSF compared to vehicle-treated rats (*p* = 0.0057 and *p* < 0.0001 respectively; Figure 1C). At 6 h postdose, there was a significant treatment effect (H (3) = 15.1, *p* = 0.005) when rats administered 10 mg/kg CY6463 (*p* = 0.004), but not 3 mg/kg CY6463 (*p* = 0.3883), had higher levels of CSF cGMP vs. vehicle-treated rats (data not shown; CSF at the 6 h timepoint was not collected in animals administered 0.1 or 1.0 mg/kg CY6463). By contrast, CSF cGMP levels in rats administered 30 mg/kg vericiguat were similar to vehicle-treated rats at 1 h (t (30) = 0.7853, *p* = 0.4384, Figure 1D) and at 6 h postdose (t (32) = 1.48, *p* = 0.1487; data not shown).

Investigating brain cGMP levels in mice after CY6463 dosing revealed that at 30 min postdose, there was a significant treatment effect (F (4,45) = 54.86, *p* < 0.001). A Dunnett’s post hoc test showed that concentrations of cGMP in the cerebrum were higher in mice treated with 3 or 10 mg/kg CY6463 vs. those treated with vehicle (*p* = 0.007 and *p* < 0.0001, respectively; data not shown). Similar levels of cGMP were measured in the cerebrum of mice treated with 0.3 or 1 mg/kg CY6463 vs. vehicle-treated mice (*p* = 0.56 and *p* = 0.40, respectively).

These data demonstrate that CY6463 is a fully CNS-penetrant compound able to elicit cGMP increases in the CNS, while vericiguat is neither CNS-penetrant nor able to elicit a cGMP elevation in the CNS.

### CY6463 Lowered Blood Pressure in Mice and Rats

Because the lowering of BP is a known pharmacodynamic effect of sGC stimulation, the effects of CY6463 on BP and HR were evaluated in rats and mice. In rats, BP effects were measured after a single administration and after multi-day dosing. In mice, BP effects were evaluated after single administration. To select appropriate doses to match BP lowering in comparative pharmacology studies, the degree of BP lowering by vericiguat was also evaluated.

#### Acute Dosing in Normotensive Rats

**FIGURE 2:**
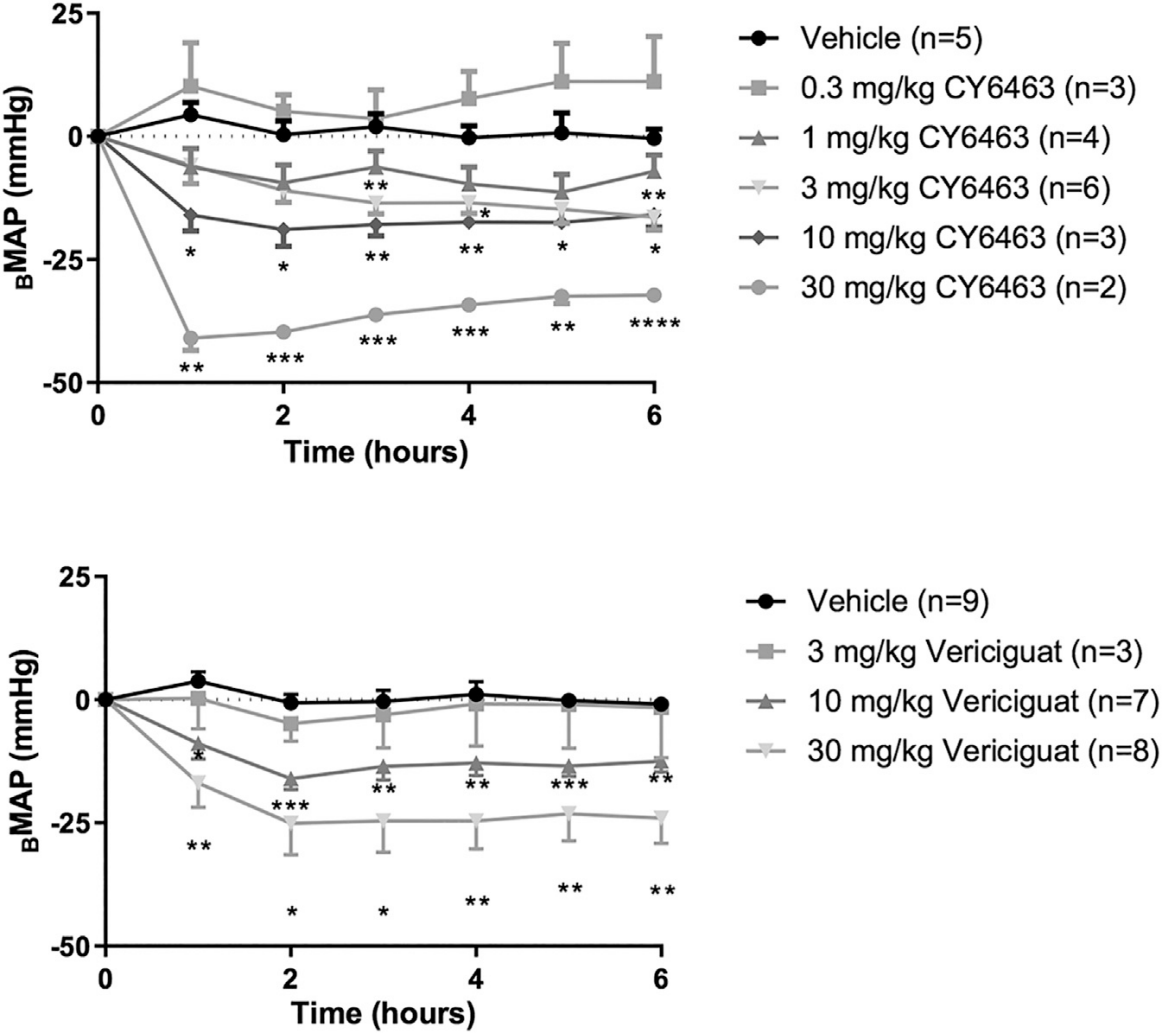
Effects of CY6463 and vericiguat on blood pressure. **(A)** Change from baseline MAP (Δ_B_MAP) in rats treated with CY6463. **(B)** Δ_B_MAP in rats treated with vericiguat. All annotated Dunnett’s post hoc tests were compared to vehicle-treated rats *p < 0.05, **p < 0.01, ***p < 0.001 and ****p < 0.0001. All data are expressed as mean ± SEM.

There was a greater decrease in MAP (as assessed by Δ_B_MAP) in rats treated with CY6463 than in vehicle-treated rats (F (5,17) = 17.66, *p* < 0.0001). Compared with vehicle-treated rats, there was a greater decrease in MAP throughout the 6 h of postdose monitoring in rats treated with 10 or 30 mg/kg CY6463, and at 3, 4, and 6 h postdose in rats treated with 3 mg/kg CY6463 but not in rats treated with 0.3 or 1 mg/kg CY6463 (Figure 2A). Peak Δ_V_MAP decreases of 45% (10- and 30-mg/kg dose groups, respectively) were observed at the C_max_ of CY6463 (1 h), whereas a peak Δ_V_MAP decrease of 16% was observed at 6 h in the 3 mg/kg CY6463 dose group. HR was unchanged by CY6463 treatment as compared to vehicle-treated rats (F (5,17) = 1.814, *p* = 0.1638; data not shown).

Rats treated with vericiguat (10 or 30 mg/kg) had a greater decrease in MAP (as assessed by Δ_B_MAP) than vehicle-treated rats (F (3,23) = 8.213, *p* = 0.0007) throughout the 6 h of monitoring (Figure 2B). Peak Δ_V_MAP was 15% lower at 2 h postdose in rats treated with 10 mg/kg vericiguat and was 26% lower in rats treated with 30 mg/kg vericiguat at 4 h postdose. At C_max_ (6 h postdose), Δ_V_MAP was 12% and 23% lower for the 10- and 30-mg/kg dose groups, respectively. HR was unchanged by vericiguat treatment as compared to vehicle treatment (F (3,23) = 1.657, *p* = 0.2040; data not shown).

#### 6-days Dosing in Normotensive Rats

To assess the effect of repeated dosing of CY6463 on BP, rats were administered 10 mg/kg CY6463 once daily for 6 days. CY6463-treated rats had a greater decrease in MAP (as assessed by Δ_B_MAP) vs. vehicle-treated rats (F (1,11 = 16.38, *p* = 0.0019) during the 4-h postdose period on all recording days; yet by 24 h after the 1^st^ and 5^th^ doses, MAP in CY6463-treated rats was not different from that in vehicle-treated rats. There was no significant difference in change in HR between rats treated with CY6463 or vehicle (F (1,13) = 2.588, *p* = 0.132; data not shown).

#### Acute Dosing in Normotensive Mice

Throughout a 6-h period after a single oral dose, there was a significant effect on MAP (as assessed by Δ_B_MAP) in mice (F (3,40) = 22.48, *p* < 0.0001; data not shown) treated with 10 mg/kg CY6463 (*p* < 0.0001) vs. vehicle-treated mice, but not in mice treated with 0.1 mg/kg (*p* = 0.819) or 1 mg/kg (*p* = 0.9995; data not shown). A peak Δ_V_MAP decrease of 19% at 1 h (*p* < 0.0001) and a 6-h average Δ_V_MAP decrease of 14% (*p* < 0.0001) was observed in mice treated with 10 mg/kg CY6463 vs. vehicle-treated mice.

Dose-responsive elevations in HR were observed in CY6463-treated vs. vehicle-treated mice (F (3,40) = 5.009, *p* = 0.0048; data not shown). HR increased up to 152 bpm (28%) from baseline 1 h after 10 mg/kg CY6463 (*p* < 0.0001) and remained elevated during the next 3 h (*p* < 0.05). The 1-mg/kg dose led to a transient increase of 88 bpm (16%) from baseline at 1 h postdose (*p* < 0.01). No change in HR was observed in mice treated with 0.1 mg/kg CY6463.

### CY6463 Elicits a Positive fMRI-BOLD Signal That is Distinctive From Vericiguat

**FIGURE 3:**
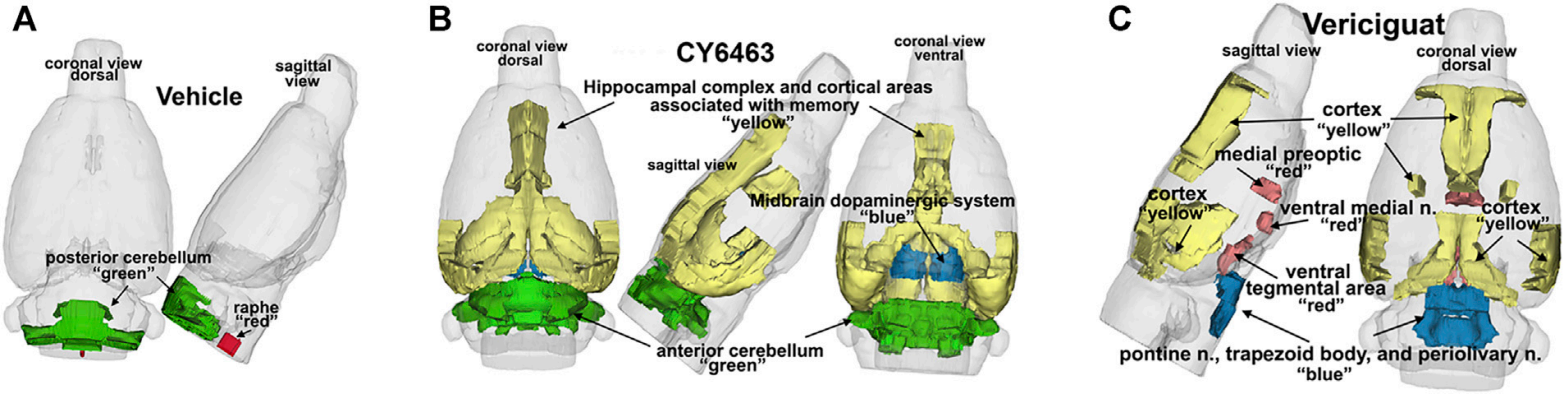
3D representation comparing CY6463 and vericiguat vs vehicle. **(A)**, positive BOLD activation for vehicle. **(B)**, positive BOLD activation for CY6463, hippocampal complex, and cortical areas associated with memory (yellow), anterior cerebellum (green), and midbrain dopaminergic system (blue). **(C)**, positive BOLD activation for vericiguat, cortex (yellow), medial preoptic, ventral medial nuclei and ventral tegmentum (red) pontine n, periolivary n, and trapezoid (blue) posterior cerebellum (green).

To determine the effects of sGC stimulation by CY6463 on fMRI-BOLD signal in the brain, rats received a single infusion of vehicle, vericiguat, or CY6463. Imaging was performed before and after infusion of the compounds. The median number of positive voxels activated for both vehicle and CY6463 at 15–25 min postdose relative to predose are shown in Table 2; shown are only those areas in CY6463- or vericiguat-treated rats that had a significant difference in positive BOLD activation and an increase relative to vehicle-treated rats across the 171 brain regions analyzed. In total, 48/171 brain areas were significantly affected by CY6463 vs. vehicle treatment, while 21/171 brain areas were affected by vericiguat. The number of positive voxels was greater after CY6463 infusion vs. vehicle in 46/48 activated regions; in contrast, the number of positive voxels after vericiguat infusion was greater vs. vehicle in 13/21 regions (Table 2). There was little activation after vehicle infusion (Figure 3A). For CY6463, these areas coalesce into major brain regions (Figure 3B), including the anterior cerebellum (1st, 2nd, 3rd, 4th, and 5th cerebellar lobules). Notably, a positive BOLD signal was observed in the deep cerebellar nuclei (dentate, interposed, and fastigial). CY6463 also selectively increased BOLD signal in the midbrain dopaminergic system (e.g., ventral tegmental area, substantia nigra compacta, and reticularis) and a joining area interpeduncular nucleus that connects with the habenula. The last major area of BOLD signal change was the hippocampal complex and surrounding cortical areas involved in memory, including the CA1, subiculum, lateral septum, entorhinal, perirhinal, ectorhinal, temporal, retrosplenial, and anterior cingulate cortices. CY6463 induced a positive BOLD fingerprint that is distinguished from vericiguat, which mainly consisted of positive BOLD signal in cortical regions (Figure 3C and Table 2).

**TABLE 2:** Median number of positive voxels activated for CY6463 vs. vehicle and vericiguat vs. vehicle.

*Brain Area*	*Vehicle*	*Vericiguat*	*p value*	*Brain Area*	*Vehicle*	*CY6463*	*p value*
***auditory ctx***	12	66	0.001	***retrosplenial caudal ctx***	4	91	0.001
***periolivary nucleus***	0	21.5	0.002	***interpeduncular nucleus***	0	10	0.001
***perirhinal ctx***	0	15.5	0.003	***habenula nucleus***	0	8	0.001
***pontine nuclei***	4.5	51	0.004	***substantia nigra reticularis***	1	21	0.002
***temporal ctx***	0.5	6	0.007	***ventral tegmental area***	0	9	0.002
***trapezoid body***	0	1.5	0.009	***pontine nuclei***	5	52	0.002
***retrosplenial caudal ctx***	4	39.5	0.011	***visual 2 ctx***	16	87	0.002
***anterior cingulate area***	3	48.5	0.019	***medial amygdaloid nucleus***	1	7	0.004
***ventral medial nucleus***	2	10.5	0.023	***substantia nigra compacta***	0	4	0.005
***secondary motor ctx***	13.5	78.5	0.026	***4th cerebellar lobule***	0	18	0.005
***supramammillary nucleus***	0	1.5	0.031	***insular ctx***	57	22	0.006
***medial preoptic area***	0	7	0.047	***5th cerebellar lobule***	1	55	0.006
***primary somatosensory ctx***	0	1	0.049	***visual 1 ctx***	11	76	0.006
***shoulder***				***interposed nucleus***	0	7	0.007
				***parietal ctx***	1	22	0.007
				***retrosplenial rostral ctx***	8	30	0.008
				***secondary motor ctx***	14	56	0.008
				***inferior colliculus***	13	31	0.009
				***1st cerebellar lobule***	0	8	0.009
				***2nd cerebellar lobule***	2	44	0.011
				***periolivary nucleus***	0	8	0.014
				***3rd cerebellar lobule***	2	34	0.015
				***subiculum dorsal***	0	8	0.015
				***pineal gland***	0	2	0.016
				***central gray***	0	4	0.016
				***superior colliculus***	5	35	0.017
				***simple lobule cerebellum***	1	62	0.018
				***ectorhinal ctx***	0	12	0.019
				***entorhinal ctx***	24	130	0.019
				***primary somatosensory ctx jaw***	49	22	0.021
				***posterior hypothalamic area***	0	11	0.023
				***medial cerebellar nucleus fastigial***	0	7	0.025
				***cortical amygdaloid nucleus***	10	25	0.025
				***medial mammillary nucleus***	1	5	0.026
				***dentate n. cerebellum***	0	2	0.028
				***perirhinal ctx***	0	8	0.029
				***rostral piriform ctx***	14	5	0.031
				***supramammillary nucleus***	0	1	0.034
				***temporal ctx***	1	15	0.037
				***flocculus cerebellum***	0	3	0.039
				***pontine reticular nucleus caudal***	0	11	0.041
				***anterior cingulate area***	3	32	0.041
				***CA1 dorsal***	0	15	0.042
				***dentate gyrus ventral***	0	4	0.046
				***basal amygdaloid nucleus***	2	12	0.046
				***lateral septal nucleus***	3	15	0.048
				***raphe magnus***	0	6	0.049
				***auditory ctx***	12	48	0.049

The table shows the median number of positive voxels activated for vehicle-, vericiguat-, and CY6463-treated rats at 20–30 min of imaging relative to the first 5-min baseline imaging (predose). The volume of activation was compared across experimental groups using the non-parametric Kruskal–Wallis test followed by a Wilcoxon rank-sum test. A *p*-value < 0.05 was considered statistically significant.

### CY6463, but Not Vericiguat, Increased Gamma-Band Power

**FIGURE 4:**
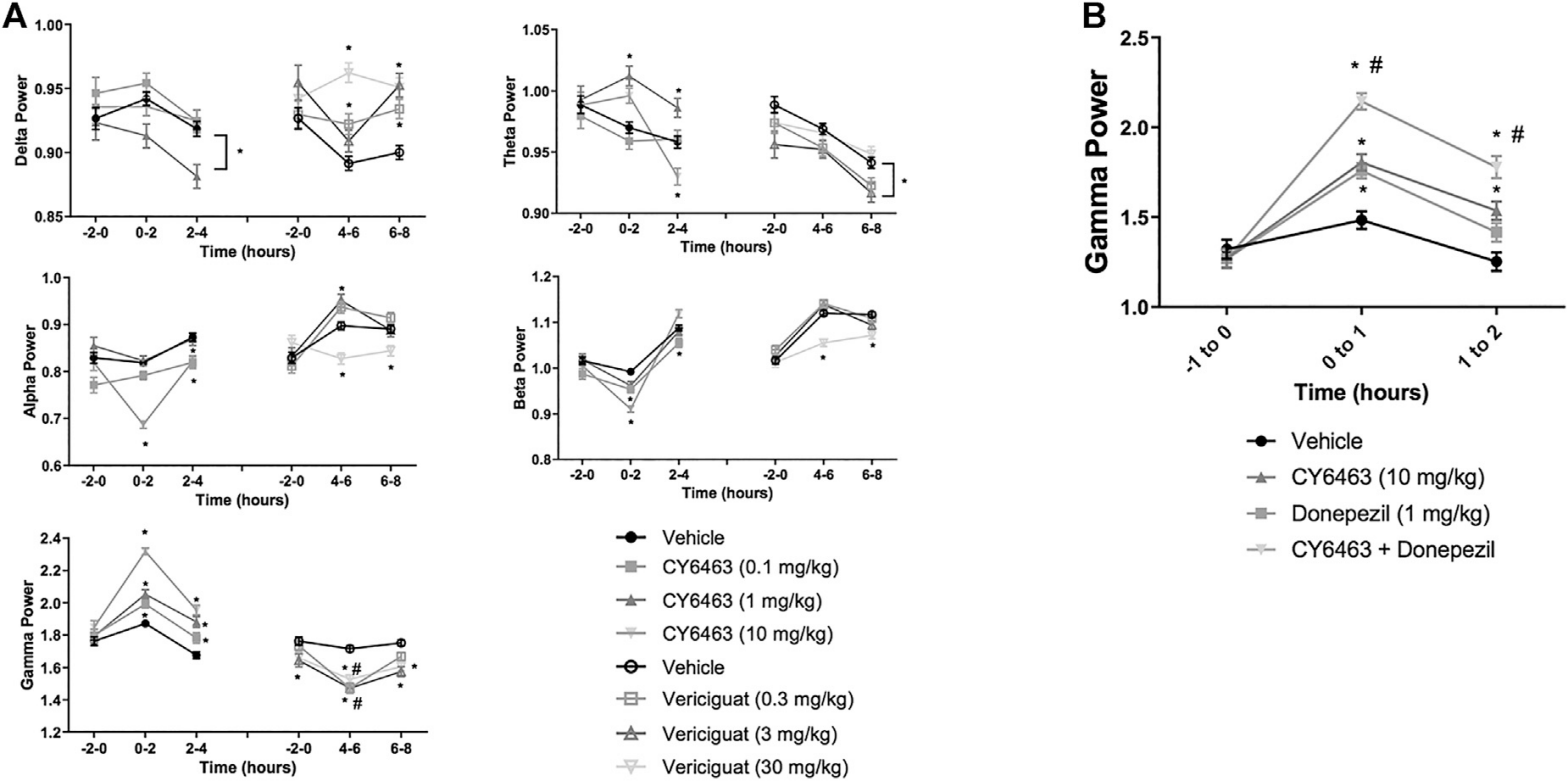
Effects of compound administration on cortical qEEG in awake during the A.M. period. **(A)**, CY6463 and vericiguat, annotated Dunnett’s post hoc tests *p < 0.05 compared to vehicle-treated rats, ^#^ *p* < 0.05 compared to baseline. **(B)**, CY6463 and donepezil. Annotated Tukey’s post hoc tests: *p < 0.05 compared to vehicle-treated rats, #*p* < 0.05 compared to CY6463. All data are expressed as mean ± SEM.

To determine the effects of a CNS-penetrant sGC stimulator on cortical oscillatory activity, the effects of CY6463 and vericiguat on relative spectral power as measured by quantitative electroencephalography were examined for 2 h predose and during the T_max_ for each of the compounds tested after the A.M. dosing (0–4 h postdose for CY6463, and 4–8 h postdose for vericiguat; Figure 4A). The same timepoints were also analyzed after P.M. dosing (data not shown). Doses were chosen to elicit similar changes in BP; thus 30 mg/kg vericiguat and 10 mg/kg CY6463 were chosen as the highest doses and then lower doses were chosen as a log lower (Figures 2A,B). Unless noted, throughout all the spectral analysis for the A.M. period, there was no effect of the group prior to compound administration. After a significant interaction, a Dunnett’s post hoc test was used to compare compound-treated rats to vehicle-treated rats within each recording time interval. If there was not a significant interaction, only the overall main effect was analyzed. Full results are displayed in Table 3. During the A.M. period when animals were awake, rats treated with any of the dose levels of CY6463 had higher gamma power vs. vehicle-treated rats throughout the recording period (Figure 4A). In contrast, gamma power was lower in rats treated with any of the dose levels of vericiguat (Figure 4A). However, in the baseline recording period, rats in the 3- and 30-mg/kg vericiguat group had lower gamma power vs. the vehicle-treated rats (*p* = 0.011 and *p* = 0.0194, respectively). Because the groups were different at baseline, a second Dunnett’s post hoc analysis was performed comparing each group to its own baseline. For vehicle-treated rats, there was no difference between 4 and 6 h postdose (*p* = 0.3645) or from 6 to 8 h (*p* = 0.9398) compared to their baseline. For all dose levels of vericiguat, gamma power was lower at 4–6 h (0.3 mg/kg, *p* < 0.0001; 3 mg/kg, *p* = 0.0002; 30 mg/kg, *p* = 0.0002) but not at 6–8 h postdose (0.3 mg/kg, *p* = 0.1442; 3 mg/kg, *p* = 0.1943; 30 mg/kg, *p* = 0.2530) compared to each group’s baseline.

**TABLE 3:** Effects of CY6463 and vericiguat on spectral band powers vs vehicle in rats.

Delta					
CY6463^a^			Vericiguat		
F (3,540) = 7.857, *p* < 0.001			F (3,524) = 15.59, *p* < 0.001		
Hours postdose	Dose	*p*-value	Hours postdose	Dose	*p*-value
0–2 and 2–4	0.1	*p* = 0.2482	4–6	0.3	↑ *p* = 0.0330
				3.0	*p* = 0.3919
	1.0	**↓** *p* = 0.0089		30	↑ *p* < 0.0001
			6–8	0.3	↑ *p* = 0.0127
	10	*p* = 0.9636		3.0	↑ *p* = 0.0002
				30	↑ *p* < 0.0001

Theta					
CY6463			Vericiguat^a^		
F (3,540) = 9.169, *p* < 0.001			F (3,540) = 7.857, *p* < 0.001		
Hours postdose	Dose	*p*-value	Hours postdose	Dose	*p*-value
0–2	0.1	*p* = 0.6451	4–6 and 6–8	0.3	↓ *p* = 0.0110
	1.0	↑ *p* = 0.0006			
	10	*p* = 0.0650		3.0	↓ *p* = 0.0001
2–4	0.1	*p* = 0.9934			
	1.0	↑ *p* = 0.0370		30	*p* = 0.8613
	10	↓ *p* = 0.0403			

Alpha					
CY6463			Vericiguat		
F (3,540) = 23.41, *p* < 0.001			F (3,524) = 8.22, *p* < 0.001		
Hours postdose	Dose	*p*-value	Hours postdose	Dose	*p*-value
0–2	0.1	*p* = 0.2842	4–6	0.3	*p* = 0.0726
	1.0	*p* = 0.9918		3.0	↑ *p* = 0.0127
	10	↓ *p* < 0.0001		30	↓ *p* = 0.0002
2–4	0.1	↓ *p* = 0.0080	6–8	0.3	*p* = 0.3811
	1.0	*p* = 0.9915		3.0	*p* = 0.9982
	10	↓ *p* = 0.015		30	↓ *p* = 0.0229

Beta					
CY6463			Vericiguat		
F (3,2334) = 6.714, *p* = 0.0002			F (3,2266) = 20.22, *p* < 0.0001		
Hours postdose	Dose	*p*-value	Hours postdose	Dose	*p*-value
0–2	0.1	↓ *p* = 0.0107	4–6	0.3	*p* = 0.1929
	1.0	*p* = 0.0596		3.0	*p* = 0.3240
	10	↓ *p* < 0.0001		30	↓ *p* < 0.0001
2–4	0.1	↓ *p* = 0.0388	6–8	0.3	*p* = 0.8091
	1.0	*p* = 0.9183		3.0	*p* = 0.1710
	10	*p* = 0.0575		30	↓ *p* = 0.0004

Gamma					
CY6463			Vericiguat		
F (3,2886) = 39.61, *p* < 0.0001			F (3,2802) = 23.6, *p* < 0.0001		
Hours postdose	Dose	*p*-value	Hours postdose	Dose	*p*-value
0–2	0.1	↑ *p* = 0.0159	4–6	0.3	↓ *p* < 0.0001
	1.0	↑ *p* < 0.0001		3.0	↓ *p* < 0.0001
	10	↑ *p* < 0.0001		30	↓ *p* < 0.0001
2–4	0.1	↑ *p* = 0.0375	6–8	0.3	*p* = 0.0738
	1.0	↑ *p* < 0.0001		3.0	↓ *p* < 0.0001
	10	↑ *p* < 0.0001		30	↓ *p* = 0.0004

The effect on spectral band power as compared to vehicle-treated rats within the indicated time interval was analyzed after a significant overall main effect and interaction. The F value for the main effect is listed. A *p*-value <0.05 was considered statistically significant. Arrows represent the direction of difference vs. vehicle-treated rats.

a If no significant interaction, then the main effect across both time intervals were analyzed.

The effect of CY6463 or vericiguat on delta, theta, alpha, and beta powers during the A.M. period when animals were awake are summarized in Table 3.

During the dark cycle (P.M. period) and for 0–2 h after dosing, gamma power increased and beta power decreased in rats treated with 10 mg/kg CY6463, but not with 0.1 or 1 mg/kg CY6463 (data not shown). During the P.M. period, the 10-mg/kg dose of CY6463 significantly decreased delta power and increased theta power. After the P.M. dose, vericiguat at 3 and 30 mg/kg increased delta power compared to vehicle and also reduced alpha, beta, and gamma power. However, some of the effects observed with vericiguat after the P.M. dosing were already noticeable during the predose period (data not shown).

### CY6463 Elicited an Additive Increase in Gamma Power When Co-administered With Donepezil

Given the effects of CY6463 on gamma oscillatory activity in the cortex, we tested the effects of CY6463 alone and in combination with donepezil on gamma power in the fronto-parietal cortex during wakefulness (active wake and quiet wake). Donepezil administration was previously reported to increase cortical gamma power in rodent models (Amat-Foraster et al., 2017). There was an overall main effect of compound treatment on gamma power (F (3,2949) = 28.7, *p* < 0.001). There was no difference among any of the groups in the predose recording period. In the first hour after dosing, donepezil, CY6463, and the combination all had higher gamma power than vehicle-treated rats (*p* = 0.0077, *p* = 0.0005, and *p* < 0.0001, respectively). In the second hour after dosing, gamma power was higher in rats treated with CY6463 or the combination than in vehicle-treated rats (*p* = 0.005 and *p* < 0.0001, respectively), but there was no longer a significant effect of donepezil monotherapy (*p* = 0.5066) (Figure 4B). At both 1 and 2 h postdose, gamma band power was greater in rats treated with the combination of CY6463 + donepezil than in rats treated with CY6463 alone (*p* < 0.0001 and *p* = 0.0295, respectively).

### CY6463 Prevented the Learning Deficit Induced by MK-801 Administration in the NOR Task in Rats

**FIGURE 5:**
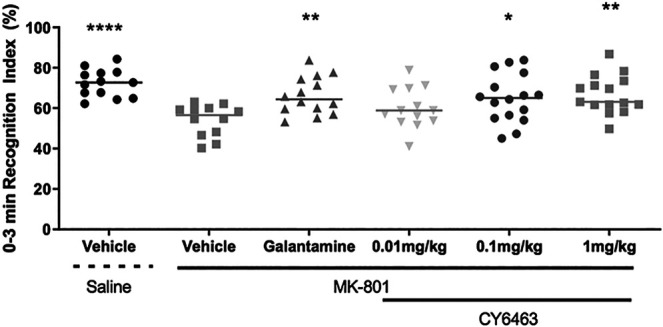
Recognition index of the 0- to 3- min interval. MK-801 caused a strong memory deficit and galantamine, 0.1 mg/kg CY6463 and 1 mg/kg CY6463 prevented the MK-801-induced memory deficits (*p < 0.05, **p < 0.01, ****p < 0.0001 vs. vehicle + MK-801).

To determine the effects of CY6463 on cognitive performance in rats, acute administration of CY6463 was evaluated in rats with a deficit in NOR induced by the NMDA receptor antagonist, MK-801 (Rajagopal et al., 2014). There was a significant main treatment effect during the 0- to 3-min postdose interval (F (5,77) = 5.512, *p* < 0.001). As expected, MK-801 caused a strong memory deficit (*p* < 0.0001), and galantamine significantly prevented the MK-801-induced memory deficits (*p* = 0.078). CY6463 attenuated MK-801-induced memory deficits at the 0.1- and 1 mg/kg dose levels (*p* = 0.02 and *p* = 0.004, respectively, Figure 5). Rats treated with 0.01 mg/kg CY6463 had a similar RI as MK-801+vehicle-treated rats (*p* = 0.3606). Similar findings were observed when analyzed across the intervals (0–1 and 0–5 min; data not shown).

### Improvement of LTP in the HD (R6/2) Mouse by CY6463

**FIGURE 6:**
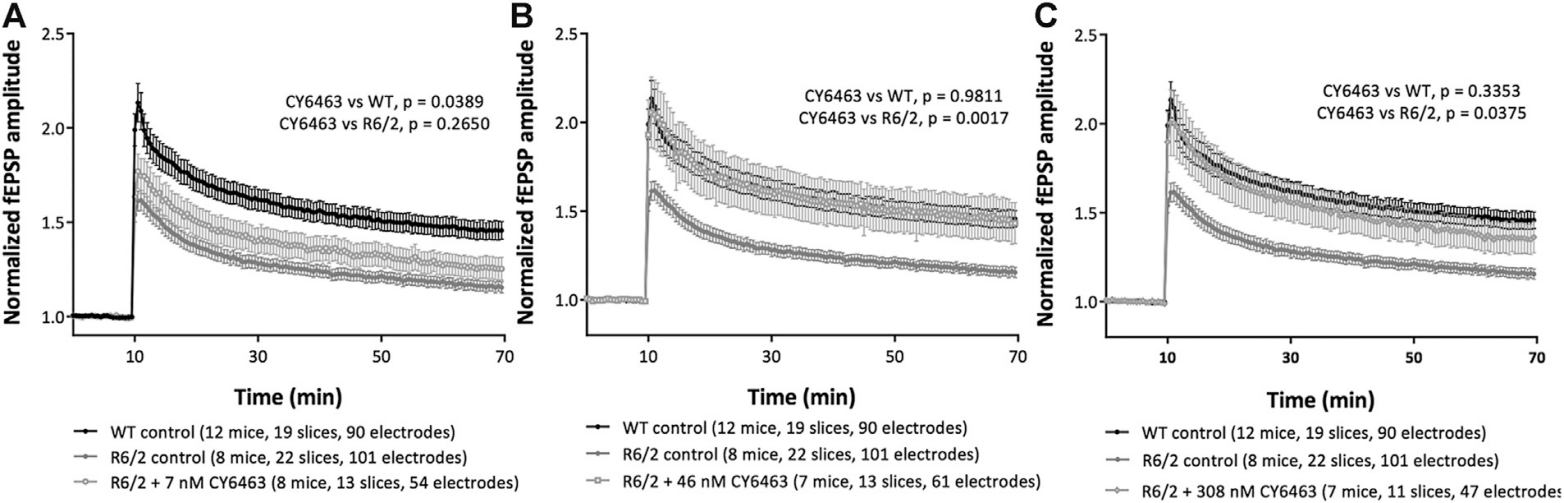
LTP in R6/2 Huntington’s mouse model. Mouse hippocampal slices in control conditions or after exposure to **(A)** 7 nM CY6463, **(B)** 46 nM CY6463, **(C)** 308 nM CY6463, and in wild-type (WT) mice hippocampal slices. The mean value of fEPSP amplitude is plotted ± SEM vs. time. The entire recording time is displayed, but only data from the last 20 min of the recording period were analyzed using Repeated Measures two-way ANOVA without Geisser-Greenhouse correction followed by an uncorrected Fisher’s LSD comparing all groups to each other. *p* values are noted in figure legends.

To determine if activation of the NO-sGC-cGMP pathway can modulate synaptic plasticity, we studied a model of neurodegeneration (R6/2 model of HD) that has an LTP deficit (Beaumont et al., 2014). We analyzed the last 20 min of recordings (30–50 min after LTP induction) in hippocampal slices from R6/2 mice pre-incubated with or without CY6463, as well as slices from WT littermate control mice. There was a significant effect of group (F (4,73) = 4.559, *p* = 0.0024): LTP was significantly impaired (*p* = 0.005) in hippocampal slices from R6/2 mice vs. age-matched WT mice (Figure 6), as previously described (Beaumont et al., 2014). In hippocampal slices from WT mice (control conditions), HFS triggered a potentiation of the evoked response amplitudes that stabilized around 45% (fEPSP were increased by 46 ± 5%, at endpoint). In hippocampal slices from R6/2 mice (control conditions), HFS triggered a potentiation of the evoked response amplitudes that stabilized around 15% (fEPSP were increased by 16 ± 3%, at endpoint). In hippocampal slices from R6/2 mice, after exposure to 7 nM CY6463, HFS triggered a potentiation of the evoked-response amplitudes that stabilized around 25% (Figure 6A; fEPSP were increased by 26 ± 6%, at endpoint), which was not significantly different than vehicle-treated hippocampal slices from R6/2 mice (*p* = 0.2650). In hippocampal slices from R6/2 mice, after exposure to 46 nM CY6463, HFS triggered a potentiation of the evoked-response amplitudes that stabilized around 45% (Figure 6B; fEPSP were increased by 44 ± 12%, at endpoint). The potentiation observed after exposure to 46 nM CY6463 was significantly larger than the potentiation recorded in vehicle-treated hippocampal slices from R6/2 mice (*p* = 0.0017). In hippocampal slices from R6/2 mice, after exposure to 308 nM CY6463, HFS triggered a potentiation of the evoked-response amplitudes that stabilized around 35% (Figure 6C; fEPSP were increased by 37 ± 9%, at endpoint), which was significantly larger than the amplitudes recorded in vehicle-treated hippocampal slices from R6/2 mice (*p* = 0.0375). Treatment with CY6463 at 46 nM or 308 nM completely restored the LTP deficit of hippocampal slice from R6/2 mice back to the LTP level measured in hippocampal slices from WT mice. The potentiation observed in hippocampal slices from WT mice and in hippocampal slices from R6/2 mice after exposure to either 46 or 308 nM CY6463 did not significantly differ (*p* = 0.9811 and 0.3353, respectively).

### CY6463 Decreased Plasma Inflammatory Markers and Increased BDNF in Obese Mice

**FIGURE 7:**
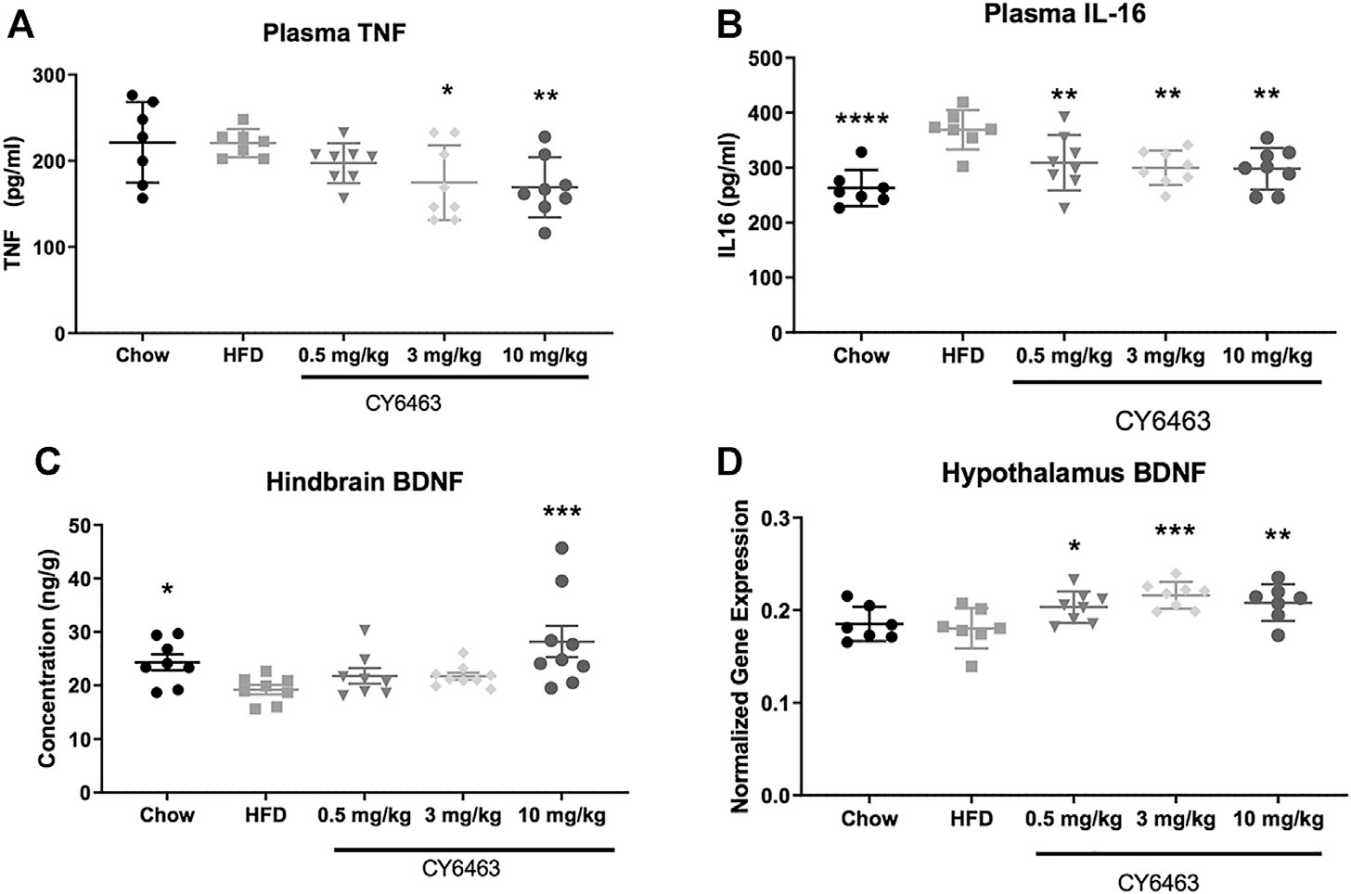
Effect of 6 weeks of treatment with CY6463 in diet-induced obese mice. **(A)**, plasma TNFα; **(B)**, plasma IL-16; **(C)**, hindbrain BDNF protein concentration; **(D)**, hypothalamic BNDF gene expression. Data were analyzed compared to HFD control mice using a one-way ANOVA followed by a Fisher’s LSD post hoc test; *p < 0.05, **p < 0.01, ****p < 0.0001. All data are expressed as mean ± SEM.

Consumption of HFD can lead to inflammation throughout the body and brain (de Git and Adan, 2015) and to decreased cognitive function (Valladolid-Acebes et al., 2011) (Baumgarner et al., 2014). Degree of inflammation can be classified by changes in gene expression and protein levels of known inflammatory markers. In this study, DIO mice were fed HFD formulated with dose levels of CY6463 for 6 weeks; plasma levels of IL-16 and TNFα were measured and BDNF gene and protein expression were quantitated. There was no significant difference in body weight (F (3,31) = 0.7414, *p* = 0.536) or food intake (F (3,31) = 0.8757, *p* = 0.464) between any of the groups that were fed HFD (data not shown). At the end of the study, plasma TNFα levels were significantly lower (F (4,33) = 3.697, *p* = 0.0136; Figure 7A) in mice receiving 3 or 10 mg/kg CY6463 than in HFD control mice (*p* = 0.016 and 0.0078, respectively). Similar TNFα levels were measured in mice treated with 0.5 mg/kg CY6463, in lean mice, and in HFD mice (*p* = 0.2063 and 0.9663, respectively). Plasma IL-16 levels in all groups were lower than in HFD control mice (F (4,33) = 6.973, *p* = 0.0003; Figure 7B). BDNF protein was measured from the hindbrain while gene expression was measured in the hypothalamus and hippocampus. In the hindbrain, BDNF protein levels were significantly higher (F (4,37) = 3.94, *p* = 0.0092; Figure 7C) in lean mice (*p* = 0.0496) and in mice treated with 10 mg/kg CY6463 (*p* = 0.0007) than in HFD control mice. In mice treated with either 0.5 or 3 mg/kg CY6463, BDNF levels were similar to HFD control mice (*p* = 0.3155 and *p* = 0.3121, respectively). There was no difference in hippocampal BDNF gene expression (F (4,33) = 0.8944, *p* = 0.4783; data not shown); yet there was a significant effect of hypothalamic BDNF gene expression (F (4,32) = 5.1, *p* = 0.0027; Figure 7D), which was higher in animals treated with CY6463 than in the HFD control and lean mice (0.5 mg/kg, *p* = 0.222; 3 mg/kg, *p* = 0.007; 10 mg/kg, *p* = 0.0079).

### CY6463 Treatment Alters Hippocampal Brain Metabolites in Aged Rats

**FIGURE 8:**
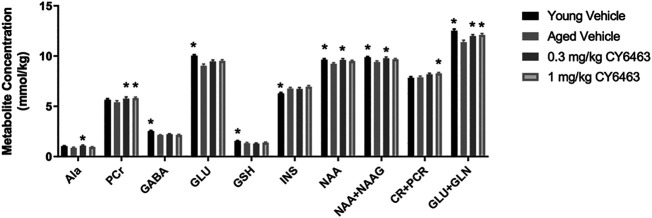
Hippocampal 1H-MRS. Young vehicle-treated and aged CY6463-treated rats vs. aged vehicle-treated rats (*p < 0.05). All data are expressed as mean ± SEM. Abbreviations for metabolites are as follows: Ala–alanine, PCr–phosphocreatine, GABA–gammaaminobutyric acid, Glc–glucose, GSH–glutathione, INS–myo-inositol, NAA–N-acetyl aspartate, N-acetyl aspartyl glutamate–NAAG.

Aging is associated with deleterious effects on the brain such as neuronal and synaptic loss and neurometabolite alterations (Paban et al., 2010). To determine if the alteration in brain metabolites that occurs during the natural process of aging could be modified by CY6463, aged rats were given dose levels of CY6463 daily for 14 days. ^1^H-MRS was then conducted to quantitate hippocampal neurometabolite levels (Figure 8). There were metabolites, alanine (Ala), phosphocreatine (PCr), and Creatine (Cr) +*p*Cr that were not different between aged and young control rats but were nonetheless higher in the aged rats treated with CY6463. There was a significant effect on Ala (F (3,37) = 3.602, *p* = 0.0222) in which young rats (*p* = 0.0688) and 1 mg/kg CY6463-treated rats (*p* = 0.5338) were not different, but 0.3 mg/kg CY6463 (*p* = 0.0117) was higher than aged vehicle-treated rats. There was a significant effect on CR + PCR (F (3,68) = 4.135, *p* = 0.0094) in which levels in young rats (*p* = 0.9799) and rats treated with 0.3 mg/kg CY6463 (*p* = 0.1394) were not different, but the level in rats treated with 1.0 mg/kg CY6463 (*p* = 0.0236) was higher than in aged vehicle-treated rats. There was a significant effect on PCr (F (3,67) = 3.069; *p* = 0.0337) in which levels in rats treated with 0.3 or 1.0 mg/kg CY6463 were higher (*p* = 0.0225 and 0.0407, respectively), while there was no difference between young and vehicle-treated aged rats (*p* = 0.4225). There were metabolites [NAA + NAAG, NAA, glutamate + glutamine (GLU + GLN)] that were higher in young vs. aged rats and on which CY6463 elicited an effect. There was a significant effect on NAA + NAAG (F (3,68) = 3.831, *p* = 0.0135) in which levels in young rats (*p* = 0.0071) and in 0.3 mg/kg CY6463-treated rats (*p* = 0.0274) were higher, but the level in the 1.0 mg/kg CY6463-treated group was not different (*p* = 0.1326) than in aged vehicle-treated rats. There was a significant effect on NAA (F (3,68) = 3.271, *p* = 0.0264) in which young rats (*p* = 0.0272) and 0.3 mg/kg CY6463-treated rats (*p* = 0.0213) were higher, but 1.0 mg/kg CY6463-treated rats (*p* = 0.1422) were not different than in aged vehicle-treated rats. There was a significant effect on GLU + GLN levels (F (3,68) = 7.94, *p* = 0.0001) in which young rats (*p* = 0.0001) and rats treated with 0.3 mg/kg (*p* = 0.0223) or 1.0 mg/kg CY6463 (*p* = 0.0053) were higher than in aged vehicle-treated rats. There was no significant effect of CY6463 on levels of Glc (F (3,63) = 2.196, *p* = 0.0973), taurine (F (3,68) = 1.506, *p* = 0.2209), creatine (Cr) (F (3,64) = 2.161, *p* = 0.1012), GLN (F (3,68) = 1.852, *p* = 0.1461), glycerophosphocholine (F (3,56) = 0.7262, *p* = 0.5406), phosphocholine (F (3,67) = 0.6876, *p* = 0.5628), lactate (F (3,37) = 2.843, *p* = 0.0509), or total cholines (F (3,68) = 1.878, *p* = 0.1415) (data not shown). There was a significant effect on gammaaminobutyric acid (GABA) (F (3,68) = 15.41,*p* < 0.0001), GLU (F (3,67) = 8.283, *p* < 0.0001), myo-inositol (INS) (F (3,68) = 5.201, *p* = 0.0027), glutathione (GSH) (F (3,68) = 3.589, *p* = 0.018) in which levels in young and aged vehicle-treated rats were different, but there was no effect of treatment with CY6463.

### Hippocampal Synaptic Loss Is Reduced by CY6463 Treatment

**FIGURE 9:**
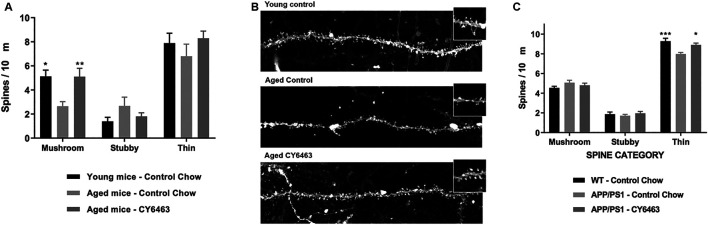
Th density of mushroom, stubby, and thin dendritic spines in the hippocampus of mouse models of neurodegeneration. **(A)**, Spine densities in the hippocampus of young mice treated with control chow, aged mice treated with control chow, and aged mice treated with chow containing CY6463. **(B)**, Representative images of hippocampal CA1 neurons apical secondary dendrites from experimental groups represented in A. **(C)**, Spine densities in the hippocampus of APP/PS1 mice treated with control chow or with chow containing CY6463, and wild-type (WT) littermate controls treated with control chow. All annotated Dunnet’s post hoc tests were performed after significant main effects or interactions in ANOVA analysis: *p,0.05, **p,0.01, ***p,0.001. All data are expressed as mean ± SEM.

Synaptic loss is a hallmark of neurodegenerative diseases (Fiala et al., 2002). The effects of CY6463 on total spine density and density of mushroom, stubby, and thin dendritic spines were evaluated in CA1 hippocampal neurons in rodent models of neurodegeneration (aged and APP/PS1 mice). We observed a significant effect of group on mushroom spine density (F (2,17) = 6.98, *p* < 0.01) in which the density was lower in aged mice treated with control chow for 18 weeks than in young mice (*p* = 0.011) (Figure 9A). However, there was no difference in mushroom spine density between aged mice treated with CY6463 vs. young mice (*p* = 0.9997). No significant differences were observed in stubby (F (2,17) = 1.66, *p* = 0.22), thin (F (2,17) = 0.91, *p* = 0.42), or total (F (2,17) = 1.444, *p* = 0.264) spine density among the groups (Figures 9A,B). Also in APP/PS1 mice, a significant effect of group for thin spines was observed (F (2,21) = 9.54, *p* < 0.001): spine density was lower in APP/PS1 mice treated with control chow than in control littermates treated with control chow for 3 months (*p* = 0.0007) and in APP/PS1 mice treated with CY6463 (*p* = 0.0123) (Figure 9C). Likewise, for total spines, there was a significant effect of group (F (2,21) = 5.164, *p* = 0.015). Total spine density was lower in APP/PS1 mice treated with control chow than in control littermates (*p* = 0.0196), and in APP/PS1 mice treated with CY6463 (*p* = 0.022). The effects observed for total spine density seem to be driven by the effects in thin spine density: density of stubby (F (2,21) = 0.51, *p* = 0.61) and mushroom (F (2,21) = 1.682, *p* = 0.21) spines were similar in APP/PS1 vs. control mice (Figure 9C).

## Discussion

sGC stimulators have the potential to treat various aspects of neurodegenerative diseases (Buys et al., 2018), yet sGC stimulators currently marketed or in clinical development do not have physicochemical properties suitable for CNS penetration (Wager et al., 2016). Our structure activity relationship optimization campaign led to the invention of CY6463, which is the first clinical-stage, orally bioavailable sGC stimulator reported to penetrate. CY6463 is bioactive in different cell types (HEK293 and rat primary neurons), and CY6463 sGC. After oral administration in rats, CY6463 is detected in the plasma and CSF and reaches distribution equilibrium between the compartments (K_p,uu_ = 0.86), which indicates passive permeability into the CNS. Compounds with these characteristics comprise the large majority of drugs approved to treat CNS disorders (Shen et al., 2004; Di et al., 2013). In contrast, vericiguat, an sGC stimulator recently approved for high-risk patients with heart failure, had a K_p,uu_ of 0.14 and is thus regarded as a non-CNS-penetrant molecule. We used vericiguat as a comparator to explore the effects of sGC stimulation by CY6463 that can be attributed to its CNS-penetrance.

As expected with the brain-penetrant nature of CY6463, oral administration of CY6463 (3 and 10 mg/kg) induced dose-dependent increases of cGMP in the CSF of rats, while 30 mg/kg vericiguat did not increase CSF cGMP levels compared to vehicle treatment. Increases of cGMP in the CSF have been described for other modulators of the NO-sGC-cGMP pathway in the brain, such as phosphodiesterase (PDE) 9 and PDE10 inhibitors, both in preclinical models (Kleiman et al., 2012) and in humans (Schwam et al., 2014; Boland et al., 2017). In addition to demonstrating target engagement in the brain, CY6463 also reduced BP in rats (3, 10, and 30 mg/kg) and mice (10 mg/kg). In these studies, no untoward effects were observed at the doses tested. BP lowering is a well-described pharmacodynamic effect of sGC stimulation due to vasodilation caused by upregulation of the NO-sGC-cGMP pathway in the vasculature (Bonderman et al., 2014; Chamorro et al., 2018) and is considered a marker of sGC target engagement. Vericiguat (10 and 30 mg/kg) reduced MAP in normotensive rats and has been reported to also lower BP in hypertensive rats (Follmann et al., 2017). After validation of target engagement in the CNS and periphery, we studied the effect of CY6463 on neuronal protection, neuronal function, and inflammation. Detriments in these domains are associated with neurodegenerative disease; therefore, improvement in any of them may lead to beneficial effects (Schliebs and Arendt, 2011; Chen et al., 2016; Sweeney et al., 2018). The approval of vericiguat for high-risk patients with heart failure highlights the value of this mechanism in cardiovascular disease. To date, there have been no studies to evaluate the efficacy of CY6463 in cardiovascular pathologies.

While both CY6463 and vericiguat reduced MAP, their effects on fMRI-BOLD signal were distinct: CY6463 increased positive fMRI-BOLD signal in 46 of 171 areas analyzed—coalescing largely in the hippocampal complex and cortical areas associated with memory, and in the anterior cerebellum and midbrain dopaminergic system—while vericiguat increased positive fMRI-BOLD signal in only 13 of 171 areas. A positive fMRI-BOLD signal is hypothesized to result from changes in CBF due to tissue demand induced by increases in neuronal activity (Gauthier and Fan, 2019). These data indicate that the CNS-penetrant CY6463 modulates CBF and neuronal activity in certain brain areas in a more pronounced way than a non-CNS penetrant sGC stimulator. The effects on fMRI-BOLD induced by vericiguat may reflect the CNS engagement due to peripheral hypotension. This study is the first demonstration of an sGC stimulator broadly affecting fMRI-BOLD signal and supports the clinical use of this non-invasive technique to explore potential CY6463 CNS pharmacodynamics.

In addition to fMRI-BOLD and neuronal activity, our data suggest that CY6463 also provides neuroprotective benefits. For instance, CY6463 increased pCREB in rat primary neurons and increased BDNF in the DIO mouse brain; CREB and BDNF are well-described signaling molecules that play a role in synaptic plasticity, neuroprotection, learning, and memory (Silva et al., 1998; Dragunow, 2004; Carlezonjr et al., 2005). Notably, the changes observed in BDNF expression were in the hypothalamus. These regions of the brain, which surround circumventricular organs, are important for nutrient signaling and metabolism and are impacted by HFD (de Git and Adan, 2015). CY6463 also had an impact in the periphery in which plasma TNFα and IL-16 levels were lower in DIO mice treated for 6 weeks with CY6463, suggesting less systemic inflammation despite remaining on the same inflammatory diet as controls. In addition, levels of hippocampal NAA, a marker that is decreased in the brain of patients with neurodegenerative diseases (Moffett et al., 2007; Kantarci, 2013), was increased by CY6463 in an aged rat model of neurodegeneration. We observed lower levels of NAA and NAA + NAAG in aged rats than in young rats, consistent with previous reports (Chen et al., 2000; Mascalchi and Vella, 2020), and aged rats treated with CY6463 had higher hippocampal NAA and NAA + NAAG than vehicle-treated aged rats. Since neurodegeneration is commonly associated with synaptic loss (Sweeney et al., 2018; Wilton et al., 2019), we evaluated the effect of chronic CY6463 treatment in aged mice and in an AD mouse model (APP/PS1 mice). In both models, dendritic spine loss in the hippocampus was lower in mice treated with CY6463 than in vehicle-treated mice. Together, these results highlight the role of sGC stimulation in the brain and indicate that CY6463 has neuroprotective properties in aged rats and mice, and in AD mice, and has anti-inflammatory effects in DIO mice.

The NO-sGC-cGMP pathway has also been implicated in LTP by modulation of pre- or post-synaptic effects and downstream activation of protein kinase G (Monfort et al., 2005; Sanderson and Sher, 2013). CY6463 restored LTP in a model of neurodegeneration (R6/2 model of Huntington’s disease), illustrating the ability of sGC stimulation to modulate and improve neuronal function. Similarly, previous studies found that a PDE9 inhibitor improved LTP in hippocampal slices (van der Staay et al., 2008; Hutson et al., 2011; Kroker et al., 2012). It would be interesting to test the effects of CY6463 in LTP in healthy rodents to understand if the compound could further improve neuronal function under normal physiological conditions.

We next hypothesized that the multi-dimensional pharmacology of CY6463, including positive effects on neuronal activity, inflammation, neuronal protection, and neuronal function may lead to improvement in cognition. Here we show that cognitive performance in the NOR task, which relies on cortical and hippocampal brain areas (Ennaceur et al., 1997; Clark et al., 2000; Grayson et al., 2015), is improved in rats treated with CY6463. In addition to this behavioral impact, CY6463 also elicited dose-dependent increases in fast oscillatory gamma-band activity in the rat cortex. In contrast, the non-CNS-penetrant sGC stimulator vericiguat suppressed gamma band activity, potentially due to a reduction in BP without concurrent CNS target engagement. Increases in gamma band oscillatory power have been associated with an increase in focus, attention, and cognitive performance (Kaiser and Lutzenberger, 2005; Rojas-Líbano and Kay, 2008; Davis et al., 2011). Thus, the increase in cortical gamma oscillatory activity by CY6463 may have contributed to the cognitive improvement observed in the NOR study (Lee et al., 2014). Further, an additive effect on increasing gamma oscillatory activity was observed when CY6463 was co-administered with donepezil, a standard-of-care AD therapy. Based on these data, it would be interesting to investigate the potential of CY6463 in combination therapies with donepezil or galantamine.

Consistent with our findings, inhibition of PDEs, resulting in an increase in CSF cGMP, has been shown to improve cognitive performance in nonclinical models (Hutson et al., 2011). However, the benefit of PDE inhibition observed in nonclinical species has not translated to clinical outcomes (Heckman et al., 2015; Prickaerts et al., 2017). It is important to note that while both PDE inhibition and sGC stimulation increase cGMP levels, PDEs modulate cGMP signaling by inhibiting its degradation and preserving the levels of cGMP produced. On the other hand, sGC stimulators are positive allosteric modulators that enhance NO-sGC-cGMP signaling by increasing the production of cGMP at the source—a mechanism that may have a more robust effect on the cGMP pathway, particularly in diseases associated with NO deficiency. Additionally, PDE inhibitors and sGC stimulators may modulate different cell types. PDEs are expressed in discrete brain areas, while sGC is expressed throughout the brain (Bollen and Prickaerts, 2012; Nagasaka et al., 2017), which may allow sGC stimulation to broadly modulate CNS physiology in the brain (Hollas et al., 2019). Perhaps most important, yet least understood, is that these mechanisms may impact different intracellular pools of cGMP. For instance, in cardiomyocytes, PDE9 inhibitors have been shown to stabilize cGMP generated from the membrane-associated natriuretic peptide receptors, whereas sGC stimulators amplify cGMP through cytoplasmic sGC (Derbyshire and Marletta, 2012; Lee et al., 2015).

Taken together, these data highlight the multiple mechanisms by which CY6463 sGC stimulation in the CNS may modulate brain physiology and improve cognition. These data support the continued clinical development of CY6463, which has completed two Phase 1 clinical studies and is initiating Phase 2 studies in CNS diseases in which NO-sGC-cGMP signaling is impacted. More broadly, these data underscore the importance of NO-sGC-cGMP in brain health/CNS function and suggest sGC stimulation is a novel and potentially paradigm-shifting approach for the treatment of diseases in which neurodegeneration is a cardinal feature.

## Data Availability

The raw data supporting the conclusions of this article will be made available by the authors, without undue reservation.

## Abbreviations

Δ_B_MAP, change from baseline MAP; Δ_V_MAP, vehicle adjusted MAP; AD, Alzheimer’s disease; Ala, alanine; A.M., ante meridiem; ANOVA, analysis of variance; BDNF, brain-derived neurotrophic factor; BOLD, blood oxygen level dependent; BP, Blood pressure; cGMP, cyclic guanosine monophosphate; CNS, central nervous system; Cr, creatine; CSF, cerebrospinal fluid; DETA-NONOate, diethylenetriamine NONOate; DIO, diet-induced obesity; EEG, electroencephalogram; EMG, electromyography; eNOS, endothelial nitric oxide synthase; FBS, fetal bovine serum; fMRI, functional magnetic resonance imaging; GLN, glutamine; GLU, glutamate; HASTE, half-Fourier acquisition single-shot turbo spin echo; HEK293, human embryonic kidney 293; HFD, high-fat diet; HR, heart rate; Kp,uu, unbound distribution coefficient; LSD, least significant difference; LTP, long-term potentiation; MAP, mean arterial pressure; Min, minutes; MRS, magnetic resonance spectroscopy; NAA, N-acetyl-aspartate; NAAG, N-acetyl-aspartate-glutamate; NMDA, N-methyl-D-aspartate; NO, nitric oxide; NOR, novel object recognition; NOS, nitric oxide synthase; pCR, phosphocreatine; pCREB, phosphorylated cAMP-response element binding; PDE, phosphodiesterase; P.M., post meridien; qEEG, quantitative electroencephalography; SEM, standard error of the mean; sGC, soluble guanylate cyclase.
