# Supplementary material for: The CNS-Penetrant Soluble Guanylate Cyclase Stimulator CY6463 Reveals its Therapeutic Potential in Neurodegenerative Diseases
Source: Front Pharmacol. 2021 May 24;12:656561. doi: 10.3389/fphar.2021.656561 (PMC8181742; doi:10.3389/fphar.2021.656561)
Supplement: Supplementary file 1 [file Table1.docx]

Supplementary Table 1: List of abbreviations and definitions.

| **Abbreviation** | **Definition** |
| --- | --- |
| AD | Alzheimer’s disease |
| Ala | alanine |
| A.M. | ante meridiem |
| ANOVA | analysis of variance |
| BDNF | brain derived neurotrophic factor |
| BOLD | blood oxygen level dependent |
| BP | Bbood pressure |
| cGMP | cyclic guanosine monophosphate |
| CNS | central nervous system |
| Cr | creatine |
| CSF | cerebrospinal fluid |
| Δ_B_MAP) | change from baseline MAP |
| Δ_V_MAP | vehicle adjusted MAP |
| DETA-NONOate | diethylenetriamine NONOate |
| DIO | diet-induced obesity |
| EEG | electroencephalogram |
| EMG | electromyography |
| eNOS | endothelial nitric oxide synthase |
| FBS | fetal bovine serum |
| fMRI | functional magnetic resonance imaging |
| GLN | glutamine |
| GLU | glutamate |
| HASTE | half-Fourier acquisition single-shot turbo spin echo |
| HEK293 | human embryonic kidney 293 |
| HFD | high-fat diet |
| HR | heart rate |
| K_p,uu_ | unbound distribution coefficient |
| LSD | least significant difference |
| LTP | long-term potentiation |
| MAP | mean arterial pressure |
| Min | minutes |
| NAA | N-acetyl-aspartate |
| NAAG | N-acetyl-aspartate-glutamate |
| NMDA | N-methyl-D-aspartate |
| NO | nitric oxide |
| NOR | novel object recognition |
| NOS | nitric oxide synthase |
| pCR | phosphocreatine |
| pCREB | phosphorylated cAMP-response element binding |
| PDE | phosphodiesterase |
| P.M. | post meridien |
| qEEG | quantitative electroencephalography |
| SEM | standard error of the mean |
| sGC | soluble guanylate cyclase |
